# Profiling the tyrosine phosphoproteome of different mouse mammary tumour models reveals distinct, model-specific signalling networks and conserved oncogenic pathways

**DOI:** 10.1186/s13058-014-0437-3

**Published:** 2014-09-09

**Authors:** Naveid A Ali, Jianmin Wu, Falko Hochgräfe, Howard Chan, Radhika Nair, Sunny Ye, Luxi Zhang, Ruth J Lyons, Mark Pinese, Hong Ching Lee, Nicola Armstrong, Christopher J Ormandy, Susan J Clark, Alexander Swarbrick, Roger J Daly

**Affiliations:** 1 Cancer Research Program, The Kinghorn Cancer Centre, Garvan Institute of Medical Research, 370 Victoria Street, Sydney, 2010 NSW Australia; 2 Current Address: Junior Research Group Pathoproteomics, Competence Center in Functional Genomics, University of Greifswald, F.-L.-Jahnstr. 15, Greifswald, 17489 Germany; 3 Current Address: Department of Biochemistry and Molecular Biology, School of Biomedical Sciences, Monash University, Level 1, Building 77, Clayton, 3800 VIC Australia; 4 School of Mathematics and Statistics, The University of Sydney, Room 637, Carslaw Building F07, Sydney, 2006 NSW Australia

## Abstract

**Introduction:**

Although aberrant tyrosine kinase signalling characterises particular breast cancer subtypes, a global analysis of tyrosine phosphorylation in mouse models of breast cancer has not been undertaken to date. This may identify conserved oncogenic pathways and potential therapeutic targets.

**Methods:**

We applied an immunoaffinity/mass spectrometry workflow to three mouse models: *murine stem cell virus-Neu*, expressing truncated Neu, the rat orthologue of human epidermal growth factor receptor 2, Her2 (HER2); *mouse mammary tumour virus-polyoma virus middle T antigen* (PyMT); and the *p53*^−/−^ transplant model (p53). Pathways and protein–protein interaction networks were identified by bioinformatics analysis. Molecular mechanisms underpinning differences in tyrosine phosphorylation were characterised by Western blot analysis and array comparative genomic hybridisation. The functional role of mesenchymal–epithelial transition factor (Met) in a subset of *p53*-null tumours was interrogated using a selective tyrosine kinase inhibitor (TKI), small interfering RNA (siRNA)–mediated knockdown and cell proliferation assays.

**Results:**

The three models could be distinguished on the basis of tyrosine phosphorylation signatures and signalling networks. HER2 tumours exhibited a protein–protein interaction network centred on avian erythroblastic leukaemia viral oncogene homologue 2 (Erbb2), epidermal growth factor receptor and platelet-derived growth factor receptor α, and they displayed enhanced tyrosine phosphorylation of ERBB receptor feedback inhibitor 1. In contrast, the PyMT network displayed significant enrichment for components of the phosphatidylinositol 3-kinase signalling pathway, whereas p53 tumours exhibited increased tyrosine phosphorylation of Met and components or regulators of the cytoskeleton and shared signalling network characteristics with basal and claudin-low breast cancer cells. A subset of p53 tumours displayed markedly elevated cellular tyrosine phosphorylation and Met expression, as well as *Met* gene amplification. Treatment of cultured *p53*-null cells exhibiting *Met* amplification with a selective Met TKI abrogated aberrant tyrosine phosphorylation and blocked cell proliferation. The effects on proliferation were recapitulated when Met was knocked down using siRNA. Additional subtypes of p53 tumours exhibited increased tyrosine phosphorylation of other oncogenes, including *Peak1/SgK269* and *Prex2*.

**Conclusion:**

This study provides network-level insights into signalling in the breast cancer models utilised and demonstrates that comparative phosphoproteomics can identify conserved oncogenic signalling pathways. The *Met*-amplified, *p53*-null tumours provide a new preclinical model for a subset of triple-negative breast cancers.

**Electronic supplementary material:**

The online version of this article (doi:10.1186/s13058-014-0437-3) contains supplementary material, which is available to authorized users.

## Introduction

It is well established that tyrosine kinase signalling pathways play major roles in breast cancer development and progression. Evidence for this includes (1) the amplification of the receptor tyrosine kinase (RTK) human epidermal growth factor receptor 2 (HER2)/avian erythroblastic leukaemia viral oncogene homologue 2 (erbB2) in approximately 25% of breast cancers [1]; (2) the presence of activating mutations in PIK3CA, encoding the p110α catalytic subunit of phosphatidylinositol 3-kinase (PI3K), in approximately 30% of cases [2]; and dysregulation of other signalling intermediates such as the non-RTK Src [3], the docking protein Gab2 [4] and the serine/threonine kinase Akt3 [5]. In addition, an array of data from cell line and mouse models of breast cancer confirm the oncogenic or tumour suppressor roles of particular tyrosine-phosphorylated signalling proteins [6]-[9]. These findings have led to the development of small-molecule– and antibody-based targeted therapies that have either entered the clinic or are currently undergoing clinical trials, including the use of trastuzumab to treat HER2-positive breast cancer [10].

Gene expression profiling has revealed that breast cancer can be subclassified into luminal A, luminal B, HER2, basal and claudin-low subtypes, which differ in terms of patient prognosis and response to therapy [11]. Basal and claudin-low breast cancers present a major clinical challenge, as their frequent triple-negative phenotype (lacking HER2, oestrogen and progesterone receptors) confers intrinsic resistance to HER2-targeted and endocrine therapy [12]. The HER2 subtype is characterised by amplification of the corresponding gene, and other breast cancer subtypes also exhibit characteristic perturbations in tyrosine kinase signalling. For example, increased expression of the RTKs Met and epidermal growth factor receptor (Egfr) are associated with basal and triple-negative breast cancers [13]-[15], and, at least in cell line models, the basal and claudin-low subtypes are characterised by a prominent Src family kinase (SFK)–governed network [16],[17]. Further characterisation of the signalling networks associated with different breast cancer subtypes may identify novel therapeutic targets and new applications for existing therapies, as well as biomarkers that aid patient stratification for therapy.

Genetically engineered mouse (GEM) models have greatly contributed to our understanding of the mechanisms and functions of particular oncogenes and tumour suppressors implicated in breast cancer, as well as biological processes involved in tumour progression [18]. These include GEM models in which normal or activated forms of *neu* (the rat homologue of HER2/erbB2) are expressed in the mammary gland [8], as well as the polyoma virus middle T antigen (PyMT) model [19]. PyMT mimics an activated RTK, localising to the plasma membrane, as well as in intracellular membranes, where it is phosphorylated on specific tyrosine residues by particular SFKs. Phosphorylation of PyMT creates binding sites for Shc (at Y250), the p85 subunit of PI3K (at Y315) and phospholipase Cγ1 (PLCγ1, at Y322), and key roles for the Y250 and Y315/Y322 sites in mammary tumourigenesis have been demonstrated [20]. Interestingly, transcript profiling has demonstrated that mouse mammary tumour virus (MMTV)-Neu and MMTV-PyMT model tumours are relatively homogeneous and exhibit gene expression similarities to human luminal-type cancers [21]. In contrast, the p53-null transplant model of mammary tumourigenesis is characterised by tumours exhibiting histological and molecular heterogeneity, as well as genetic instability [22],[23]. Indeed, transcript profiling has demonstrated that p53-null tumours can be classified into basal-like, luminal and claudin-low subtypes characterised by distinct genomic copy number changes [22].

An important concept emanating from research using GEM models is that comparative genomic and transcriptomic strategies, wherein particular mouse tumours are compared to human breast cancer subtypes, can be utilised identify conserved mechanisms essential for disease development and progression [21],[22],[24],[25]. In this study, we undertook global tyrosine phosphorylation profiling of three GEM models of breast cancer: a HER2 model featuring expression of an activated form of the receptor lacking the extracellular domain [26], as well as the MMTV-PyMT and p53-null transplant models. This enabled characterisation of the tyrosine phosphorylation-based signalling networks characteristic of each tumour type, revealed similarities and differences between these tumour models, and identified oncogenic pathways conserved in the human disease.

## Methods

### Plasmids

Neu-pMIL was used to generate the HER2 breast cancer model. This expresses a truncated form of Neu (approximately 647 to 1,260 amino acids, the rodent orthologue of Her2) with elevated activity due to truncation of the extracellular domain [27]. Truncated Neu was subcloned from Neu-pLJ by digestion using Sal1 followed by subcloning into pENTR2B (Invitrogen, Mulgrave, Victoria, Australia). The gene was subsequently cloned into pMIL, a derivative of pMig [28] that expresses a luciferase marker, to generate Neu-pMIL, in which expression of Neu is driven by the *murine stem cell virus* (MSCV) promoter. The Gateway LR recombination reaction was used as per the manufacturer's instructions (Invitrogen).

### Generation of tumours

#### MSCV-Neu (HER2) tumours

Primary mammary epithelial cells from FVB/N mice were cultured and retrovirally transduced with truncated Neu encoded by Neu-pMIL as described previously [26],[29],[30]. Cells were transplanted into the cleared mammary fat pad of naïve FVB/N recipients within 2 days of retroviral transduction.

#### Tp53-null tumours

*Tp53-*null (p53) mice from The Jackson Laboratory (stock no. 002899; Bar Harbor, ME, USA) were on an FVB/N background. Mammary epithelium from 8-week-old mice was transplanted to the cleared mammary fat pad of naïve BL/6 (clone 5101) or FVB/N (all other clones) recipients as described previously [16],[31].

Mice were aged for up to 12 months to permit spontaneous tumour formation [26]. Tumour fragments were passaged into the cleared mammary fat pad of naïve mice as previously described [26] (Additional file 1: Figure S1).

#### PyMT-transformed tumours

PyMT tumours were derived from MMTV-PyMT-transgenic mice on an FVB/N background as previously described [32]*.* All animal work was approved by the animal ethics committee of Garvan Institute of Medical Research, St Vincent's Hospital.

### Phosphotyrosine peptide enrichment

Resected mouse mammary tumours were lysed in 8 M urea, 20 mM 2-[4-(2-hydroxyethyl)piperazin-1-yl]ethanesulfonic acid (HEPES), 2.5 mM sodium pyrophosphate, 1 mM β-glycerol phosphate, 1 mM sodium orthovanadate, 1 mM ethylenediaminetetraacetic acid and 1 mM Tris(2-carboxyethyl)phosphine, pH 8.0. Lysates were cleared by sonication and centrifugation prior to protease digestion, phosphotyrosine (pY) immunoprecipitation (IP) and nano-liquid chromatography tandem mass spectrometry (nano-LC-MS/MS). Lysates were quantitated, and 20 mg of each sample were diluted to a final concentration of 1 M urea with 20 mM HEPES and digested overnight with trypsin at room temperature. Heavy proline (+6 Da)– and alanine (+4 Da)–labelled synthetic standard pY peptides of MK14, elongation factor Tu and EGFR were spiked into each sample at 5 pmol, 500 fmol and 10 pmol, respectively, to enable normalisation of label-free quantitative values. Peptides were then desalted and concentrated using C_18_ Sep-Pak columns (Waters, Milford, MA, USA), and lyophilised. pY peptides were subject to IP using the PhosphoScan procedure (Cell Signaling Technology, Beverly, MA, USA) with pY100 antibodies as previously described [16]. pY-enriched peptides were dried using a vacuum centrifuge and stored at −80°C prior to nano-LC-MS/MS analysis. All samples were analysed by nano-LC-MS/MS using two technical replicates.

### Nano–liquid chromatography tandem mass spectrometry

pY peptides were resuspended in 15 μl of MS buffer containing 1% formic acid, 2% acetonitrile (ACN) and 0.05% heptafluorobutyric acid and subjected to nano-LC-MS/MS. Peptides were separated by nano-LC using an UltiMate 3000 high-performance liquid chromatography and autosampler system (Dionex, Sunnyvale, CA, USA). Samples were concentrated and desalted onto a C_18_ micro-precolumn (500 μm × 2 mm; Michrom Bioresources, Auburn, CA, USA) with H_2_O:ACN (98:2, 0.05% trifluoroacetic acid) at 15 μl/min for 4 minutes. The micro-precolumn was then switched online with a nano-C_18_ column (75 μm × about 10 cm, 5 μm, 200 Å Magic; Michrom) and the reverse phase nano-eluent was subjected to positive nanoflow electrospray analysis in information-dependent acquisition (IDA) mode (250 nl/min for 30 minutes). Mass spectra were acquired on an Orbitrap Velos mass spectrometer (Thermo Electron, Waltham, MA, USA). In IDA mode, survey scans in the mass-to-charge ratio (*m*/*z*) range 350 to 1,750 were acquired with lock mass enabled. Up to the 15 most abundant ions (>5,000 counts) with charge states greater than +2 were sequentially isolated and further subjected to MS/MS fragmentation within the linear ion trap using collisionally induced dissociation. MS/MS spectra were accumulated with an activation time of 30 milliseconds at a target value of 30,000 ions and a resolution of 60,000. *m*/*z* ratios selected for MS/MS were dynamically excluded for 30 seconds.

### Protein identification

The nano-LC-MS/MS .raw files were processed with MaxQuant software (version 1.1.1.25), which uses the Andromeda algorithm for data processing, database searching and protein identification [33]. Extracted peak lists were searched against the UniProtKB/Swiss-Prot *Mus musculus* database (Version 2010_10) containing 35,052 entries (including common contaminants) and a proportionally sized decoy database for false discovery rate (FDR) generation. The following search parameters were selected: fixed cysteine carbamidomethylation modification, variable methionine oxidation modification, variable protein N-acetylation, and variable phosphorylation of serine, threonine and tyrosine. A minimum peptide length of six amino acids and up to two missed cleavages were allowed. The initial first search mass tolerance was 20 ppm for precursor ions and 0.5 Da for fragment ions. Matches between runs were enabled with default settings and label-free quantitation enabled. The FDR was limited to 1% for both protein and peptide identification. For identification of the standard peptides, additional variable modifications of heavy proline (+6 Da) and alanine (+4 Da) were enabled.

### Phosphotyrosine peptide quantitation

Raw pY peptide spectral intensities were extracted from the 'Evidence' output files generated in MaxQuant. These intensities were then normalised against pY peptide intensities for the heavy-labelled spiked-in peptide standards. These normalised intensities were then log_10_-transformed prior to all bioinformatics analyses, and their direct comparison enabled the relative quantitation of pY sites between the p53, PyMT and HER2 GEM models of breast cancer.

### Bioinformatics

Raw MaxQuant output files were subjected to the following filtering criteria prior to their inclusion in further bioinformatics analysis: (1) contaminants and reversed matches were removed, ensuring that all identifications were reported with an FDR of <1%; and (2) pY sites were filtered for a minimum localisation probability confidence of 0.75.

#### Imputation

The *k*-nearest neighbour [34] method was applied to impute missing values if a pY site was present in more than 50% of all samples; otherwise, the minimal value across all samples or the overall mean per sample for that pY site was used to replace missing values.

#### Clustering and heat map generation

Heat maps were generated using the R package *gplots* with hierarchical clustering applied to generate dendrograms for pY sites and samples. Euclidean distance with complete linkage was used, and the values were standardised at rows. The Bioconductor package *limma* was used to identify differences between the cancer models based on their pY-site expression levels. Empirical Bayes moderated *P*-values for contrasting and comparing expression between all samples were corrected for multiple testing by the Strimmer method as implemented in the R package fdrtool [35]. For all tests, *Q*-values less than 0.05 were considered significant. pY sites were thought of as class-specific (Her2-specific, p53-specific, PyMT-specific) on the basis of their contrast significance and the exhibition of increased spectral intensity for a particular sample.

#### Random forest classifier

The random forest classifier [36] was built using a two-step approach with the R package *randomForest* 2.15.0. In the first step, a classifier was built using all 381 consistently identified pY sites (that is, ≥75% of samples) as variables. In the second step, 17 pY sites with the importance score > 1.2 for each tumour model, which were calculated from the first classifier using a permutation-based approach, were used to build a new classifier. The out-of-bag estimate of error rates is 4.76% for both classifiers.

#### Network analysis

The protein–protein interaction data were extracted from Protein Interaction Network Analysis (PINA) [37],[38]; substrate–kinase relationship information was obtained from PhosphoSitePlus [39]; and mouse-human orthologues extracted from MSOAR [40]. The networks were generated using a Cytoscape PINA4MS plugin (HC Lee, M Pinese, L Bourbon, I Rooman, RJ Daly, AV Biankin, J Wu, manuscript under preparation. URL: http://apps.cytoscape.org/apps/pina4ms).

#### Pathway analysis

The KOBAS web server [41],[42] was applied for pathway enrichment analysis. The hypergeometric test was used to calculate the statistical significance of each pathway, followed by multiple test correction using the Benjamini–Hochberg method [43].

### Antibodies and immunoblotting

Antibodies against the following proteins were used, with immunoblotting carried out as previously described [44]: IRS1 (Upstate Biotechnology, Lake Placid, NY, USA); IRS1 pY612 (Invitrogen), Stat3, Crkl, Jak-1, P85α, CDK16, Cav1, ErbB3, phospho-ErbB3 (Tyr1328; Tyr1325 in mouse), ErbB2, Met, Met pY1234/1235 and pY (P-Tyr-100) (Cell Signaling Technology); phospho-p85 (Tyr467) (GeneTex, Irvine, CA, USA); platelet-derived growth factor receptor α (PDGFRα) and β-actin (Santa Cruz Biotechnology, Santa Cruz, CA, USA); ERBB receptor feedback inhibitor 1 (Errfi1) (Sigma-Aldrich, St Louis, MO, USA).

### DNA extraction

Mouse tumour samples and liver tissue samples were harvested, snap-frozen and processed using the QIAamp DNA Mini Kit (QIAGEN, Valencia, CA, USA) according to the manufacturer's instructions. Briefly, up to 25 mg of frozen tissue was ground and digested with proteinase K overnight at 56°C. DNA was purified on a QIAamp spin column, eluted in 200 μl of water and assessed for quality using a NanoDrop spectrophotometer (Thermo Scientific, Waltham, MA, USA). Samples were cleaned up by sodium acetate/ethanol precipitation to ensure 260/280 and 260/230 absorbance ratios >1.8. DNA integrity was checked using gel electrophoresis.

### Array comparative genomic hybridisation

Array comparative genomic hybridisation (aCGH) was performed by The Ramaciotti Centre for Gene Function Analysis (Randwick, NSW, Australia) on differentially labelled tumour DNA (Cy5) and sex-matched reference liver DNA (Cy3) using the Agilent SurePrint G3 mouse CGH 4 × 180 K microarray platform (Agilent Technologies, Santa Clara, CA, USA). The aCGH data were analysed using circular binary segmentation [45] to translate intensity measurements into regions of equal copy numbers. The raw array data were median-normalised; duplicate probes were averaged; and the resulting data were smoothed to remove outliers prior to segmentation. The gain and loss status for each region was assigned by determining plateaus in an ordered plot of all segments by their mean log_2_ ratios. By this method, we denoted deletions to be segments with a mean value less than −0.5, amplifications to have mean value between 0.5 and 1.5 and highly amplified regions to have a mean value greater than 1.5. Using this information, we merged segmental values across the genome to create a common set of copy number levels for each individual tumour. Analysis was carried out using the Bioconductor DNAcopy package [46] in R Project R2.15.2 software [47].

### Met inhibitor experiments

Tumours were harvested and cellular preparations made using mechanical disruption and collagenase digestion as described previously [30]. Cells were cultured *in vitro* for two passages in mouse mammary epithelial cell media consisting of Dulbecco's modified Eagle's medium/Ham's F12 nutrient mixture supplemented with foetal calf serum, mouse epidermal growth factor, hydrocortisone, HEPES, insulin, gentamicin and penicillin/streptomycin [30], then trypsinised and seeded into 96-well plates at a density of 6,000 cells/well. The following day, cells were treated with the Met inhibitor PHA-665752 (Selleck Chemicals, Houston, TX, USA) at concentrations of 0.25 μM, 0.5 μM and 1 μM or vehicle treated with 0.1% dimethyl sulphoxide (DMSO; Sigma-Aldrich). Cell proliferation was assessed and recorded using the IncuCyte ZOOM system (Essen BioScience, Ann Arbor, MI, USA). Briefly, cells were cultured for 5 days while images of cells inside each well (three images per well across four replicate wells) were captured every 2 hours. Proliferation was measured at each time point as a percentage of confluence, as determined using the IncuCyte software on the basis of automated analysis of captured images. Data are representative of two replicate experiments. For immunoblotting studies, cells at 70% to 90% confluence were treated with either vehicle control (DMSO) or Met TKI (1 μM final concentration) for 1 hour and then lysed in 1% (vol/vol) Triton X-100 lysis buffer as previously described [48].

### Met knockdown and cell proliferation assay

Met knockdown was performed using either individual (ON-TARGET*plus* Met siRNA 1 and 2) or SMARTpool siRNA targeting mouse Met (Dharmacon, Lafayette, CO, USA). The ON-TARGET*plus* nontargeting pool was used as a negative control. Briefly, cells were seeded into 96-well plates at a density of 4,500 cells/well 16 hours prior to transfection with 20 nM of siRNA using Lipofectamine 2000 reagent (Invitrogen). Cell proliferation was assessed by MTS assay ([3-(4,5-dimethyl-2-yl)-5-(3-carboxymethoxyphenyl)-2-(4-sulfophenyl)-2H-tetrazolium, inner salt; Promega, Madison, WI, USA), according to the manufacturer's protocol, 72 hours posttransfection.

## Results

### Tyrosine phosphorylation profiling of mouse mammary tumour models

**Figure 1:**
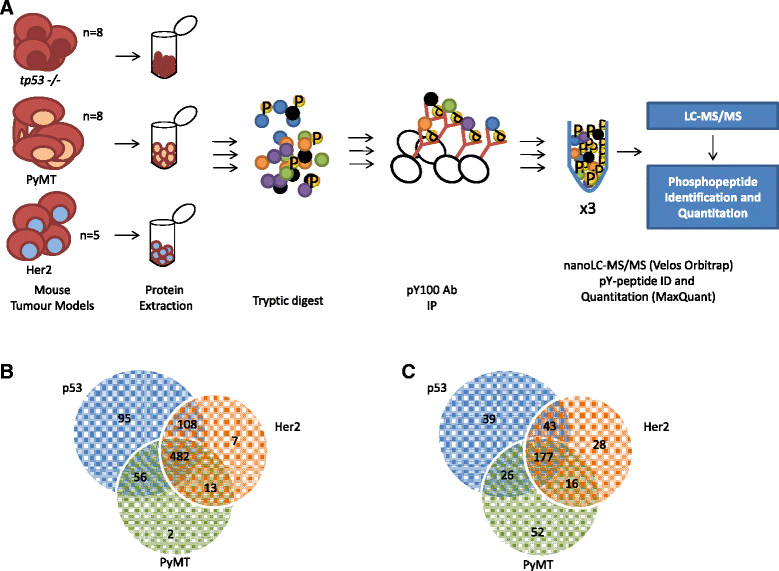
**An overview of the workflow and mass spectrometric phosphotyrosine profiling of mouse tumours. (A)** Schematic of the workflow in this study. Ab, Antibody; IP, Immunoprecipitation; nanoLC-MS/MS, Nano-liquid chromatography tandem mass spectrometry; PyMT, Polyoma virus middle T antigen. **(B)** Distribution and overlap of the 763 phosphotyrosine (pY) sites identified in the three mouse tumour models. **(C)** Distribution and overlap of the 381 highly reproducible pY sites identified in the three mouse tumour models.

Because the mammary gland develops postnatally, murine mammary epithelium can be transferred into the surgically cleared fat pads of juvenile isogenic naïve recipients. The use of GEM as donors, or viral transduction of cells after extraction, allows the rapid generation of chimeric models in which implanted transgenic or gene-knockout mammary epithelium develops in an otherwise wild-type host [21],[26]. Exploiting this strategy, we performed phosphoproteomic profiling using a combined immunoaffinity/LC-MS/MS approach across multiple MSCV-Neu (HER2), *Tp53-null* (p53) and PyMT tumours, as illustrated in Figure 1A. The lineage of each tumour from its original clone is outlined in Additional file 1: Figure S1A. In total, 856 unique pY sites were identified and quantitated from the tumour sample population with a FDR <1%. Following high-confidence localisation site filtering at ≥0.75, 763 unique pY sites from 506 unique phosphoproteins remained, with approximately 63% being common across all tumour types (Figure 1B and Additional file 2: Table S1).

These pY sites mapped to 506 unique proteins. These proteins, which were grouped based on Gene Ontology functional categories, are illustrated in Additional file 3: Figure S2A. Additional file 3: Figure S2B shows an expanded section of the kinases identified in the three tumour types. The largest proportion of kinases comprised serine/threonine kinases, followed by non-RTKs and RTKs. The least represented group comprised the dual-specificity kinases. The total pY site intensities were determined for the non-RTKs, which are shown in Additional file 3: Figure S2C. The most intense signal from the pY sites of non-RTKs was derived from SFKs (Yes, Fyn, Lyn and Hck), followed by focal adhesion kinase 1. When this approach was applied to RTKs, the most intense signal was derived from Met, followed by EphA2 (Additional file 3: Figure S2D).

To compare tyrosine phosphorylation patterns between the different tumour types and identify potential similarities, we undertook unsupervised hierarchical clustering. This revealed that two p53 tumour samples formed a distinct subgroup characterised by markedly elevated tyrosine phosphorylation of many pY sites (Additional file 4: Figure S3). The remaining tumour samples were distributed over a second major subgroup and exhibited a complex pattern of relationships. Of note, five PyMT tumours were closely related, and the majority of the p53 and HER2 tumours clustered together (Additional file 4: Figure S3). Also, although some tumours of shared origin, such as p53 tumours 1203 and 1204, clustered together, others, such as HER2 tumours HER 1197 and 3051, were well separated (Additional file 1: Figure S1B), indicating that divergence can occur following serial transplantation.

To generate a list of pY sites characteristic of each tumour type, we first identified pY sites detected in at least 75% of the corresponding samples. Therefore, we selected pY sites that were identified in at least (1) six of eight p53 tumour samples, (2) six of eight PyMT tumour samples or (3) four of five Her2 tumour samples. Following this selection-filtering process, 381 pY sites remained (Figure 1C and Additional file 5: Table S2). These sites mapped to 256 proteins, with 177 of them being found in all three tumour types. Upon unsupervised hierarchical clustering of this refined data set, all of the PyMT tumours were clustered together (Additional file 6: Figure S4). However, p53 tumours 1203 and 1204 still formed a distinct subgroup, and the HER2 tumours were divided into two groups, one related to PyMT tumours and the other to p53 tumours (Additional file 6: Figure S4).

**Figure 2:**
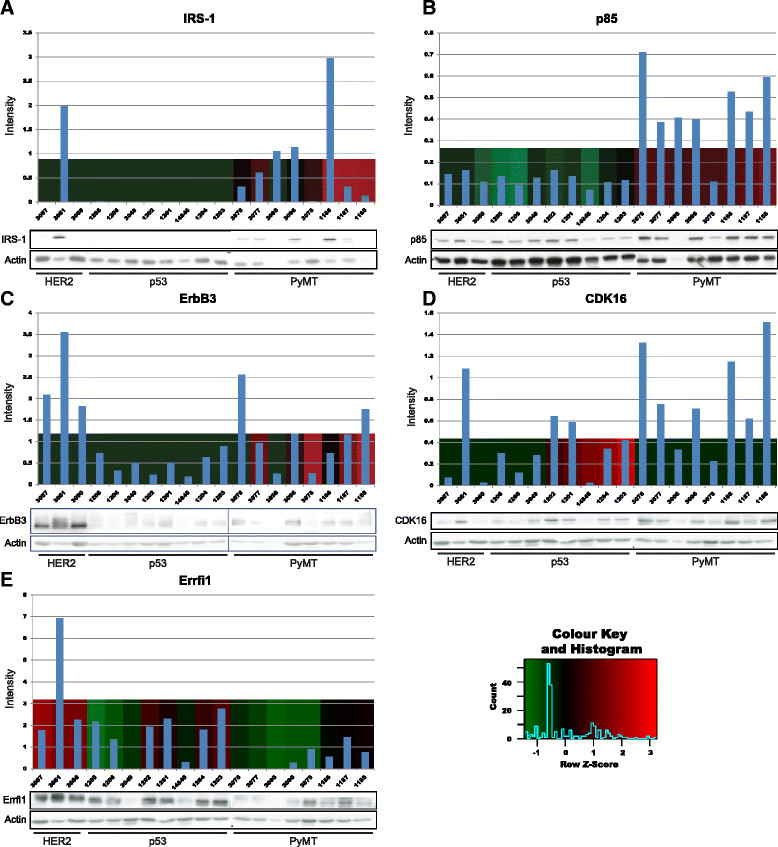
**Comparison of expression and tyrosine phosphorylation of specific proteins.** Immunoblot quantitation of total protein expression compared with relative tyrosine phosphorylation of the corresponding protein, obtained by mass spectrometry (MS). Green represents low phosphotyrosine (pY) peptide abundance as measured by MS, and red indicates high pY peptide abundance. The heat maps indicate the intensity of phosphorylation on the following specific sites: **(A)** IRS-1 (pY608), **(B)** p85 (pY467), **(C)** ErbB3 (pY1325), **(D)** CDK16 (pY176) and **(E)** Errfi1 (pY393). Blue bars represent actin-normalised immunoblot intensities for the proteins indicated. PyMT, Polyoma virus middle T antigen.

To further refine the list of sites upregulated in a particular tumour type versus the other two, an analysis of variance (ANOVA)–based approach was implemented. Thirty-two pY sites were upregulated in HER2 tumours, eighty-six in p53 tumours and eighty in PyMT tumours (Additional file 5: Table S2). Of note, certain proteins exhibited site-selective variation in phosphorylation between different tumour types, examples being Tln1 and Cbl. Elevated levels of Erbb3 pY1325 and p85 pY467 in PyMT tumours were confirmed by Western blot analysis with appropriate phosphospecific antibodies (Additional file 7: Figure S5). Immunoblotting for selected targets revealed that, for certain pY sites characteristic of a given subtype, elevated phosphorylation was accompanied by increased expression of the given protein. This was exemplified by Irs1 and Pik3r1 (p85) in PyMT tumours (Figure 2A and B). However, for others, the increased phosphorylation must reflect a change in relative phosphorylation, as for Erbb3 in PyMT tumours, Cdk16 in p53 tumours and Erffi1 in HER2 tumours (Figure 2C to E). References for the sites characterised in this figure are provided in PhosphoSite [49].

**Figure 3:**
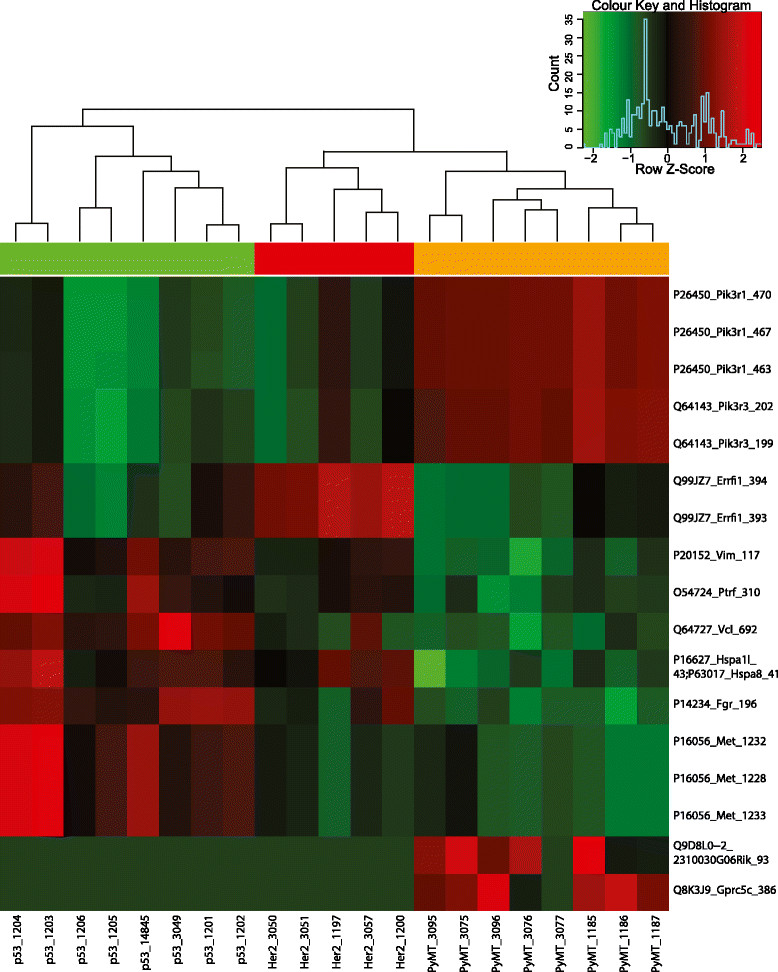
**Unsupervised hierarchical clustering using the 17--pY site** **tumour type classifier identified using random forest classification.**

We also developed and applied a random forest classifier to identify 17 highly variable pY sites that can be used to distinguish between the tumour types (Figure 3). Amongst these, elevated phosphorylation of Pik3r1, Pik3r3, a G protein–coupled receptor (Gprc5c) and an uncharacterised protein (2310030G06Rik) was specific to PyMT tumours, and p53 tumours were characterised by increased phosphorylation of Vim, Ptrf, Vcl, Fgr, Met and a protein related to heat shock protein 70 kDa. Elevated tyrosine phosphorylation of Errfi1 characterised HER2 tumours.

### Pathway and protein–protein interaction analysis

Lists of pY sites enriched in each tumour type were then subjected to bioinformatics analysis to identify associated pathways or biological processes (Table 1). Strikingly, marked differences were observed between p53, HER2 and PyMT tumours. The former exhibited an enrichment of processes linked to cytoskeletal reorganisation, with the top five significant terms being leucocyte transendothelial migration, bacterial invasion of epithelial cells, regulation of actin cytoskeleton, focal adhesion and muscle contraction. The only term significantly enriched for HER2 tumours was the ErbB signalling pathway, whereas the PyMT tumours were characterised by processes involving PI3K signalling, including type 2 diabetes mellitus, the FcεRI signalling pathway, acute myeloid leukaemia, Fcγ receptor–mediated phagocytosis and the neurotrophin signalling pathway.

**Table 1:** **Top ten pathways/biological processes for each tumour type** ^**a**^

Term	Corrected*P*-value	Accession	Gene name
**p53**			
**Leucocyte transendothelial migration**	**7.87E-05**	P70460|Q61140|P26040|P26041|Q64727|Q8VI36|P57780|Q7TPR4|P09055	Vasp|Bcar1|Ezr|Msn|Vcl|Pxn|Actn4|Actn1|Itgb1
**Bacterial invasion of epithelial cells**	**<0.01**	P22682|Q61140|Q64727|Q8VI36|Q9JM76|P49817|P09055	Cbl|Bcar1|Vcl|Pxn|Arpc3|Cav1|Itgb1
**Regulation of actin cytoskeleton**	**<0.01**	Q61140|P26040|P26041|Q7TPR4|Q64727|Q8VI36|P26043|P57780|Q9JM76|P09055	Bcar1|Ezr|Msn|Actn1|Vcl|Pxn|Rdx|Actn4|Arpc3|Itgb1
**Focal adhesion**	**<0.01**	P70460|P26039|Q61140|Q64727|Q8VI36|P57780|Q7TPR4|P49817|P09055	Vasp|Tln1|Bcar1|Vcl|Pxn|Actn4|Actn1|Cav1|Itgb1
**Muscle contraction**	**<0.01**	P62204|Q64727|P20152|Q8VI36|P26039	Calm1|Vcl|Vim|Pxn|Tln1
**Haemostasis**	**<0.01**	P12023|P20491|P26039|Q61140|Q91YI4|P14234|P48025|P57780|Q7TPR4|P49817|P09055	App|Fcer1g|Tln1|Bcar1|Arrb2|Fgr|Sykb|Actn4|Actn1|Cav1|Itgb1
**Fcγ receptor–mediated phagocytosis**	**0.02**	Q9JM76|P70460|P08103|Q9ES52|P48025	Arpc3|Vasp|Hck|Inpp5d|Sykb
**Adherens junction**	**0.05**	Q9D358|P57780|Q7TPR4|Q64727	Acp1|Actn4|Actn1|Vcl
**Cell–cell communication**	**0.05**	Q80W68|Q9QXS1|Q7TPR4|P09055|Q3UND0	Kirrel|Plec|Actn1|Itgb1|Skap2
Endocytosis	0.12	P22682|Q91YI4|P01899|Q99LI8|Q62351|P49817	Cbl|Arrb2|H2-D1|Hgs|Tfrc|Cav1
**Her2**			
**ErbB signalling pathway**	**3.79E-02**	P70424|Q01279|P47941|P22682|Q62077	Erbb2|Egfr|Crkl|Cbl|Plcγ1
Disease	0.09	Q99LI8|P35235|Q01279|P22682|Q62077	Hgs|Ptpn11|Egfr|Cbl|Plcγ1
Non–small-cell lung cancer	0.16	P70424|Q01279|Q62077	Erbb2|Egfr|Plcγ1
Pathways in cancer	0.16	P22682|Q62077|P47941|Q01279|P26618|P70424|P97807	Cbl|Plcγ1|Crkl|Egfr|Pdgfrα|Erbb2|Fh1
Glioma	0.16	Q62077|Q01279|P26618	Plcγ1|Egfr|Pdgfrα
Focal adhesion	0.16	P70424|Q01279|P47941|P26039|P26618	Erbb2|Egfr|Crkl|Tln1|Pdgfrα
Renal cell carcinoma	0.16	P35235|P47941|P97807	Ptpn11|Crkl|Fh1
Neurotrophin signalling pathway	0.16	O08911|P35235|P47941|Q62077	Mapk12|Ptpn11|Crkl|Plcγ1
Chronic myeloid leukaemia	0.16	P35235|P47941|P22682	Ptpn11|Crkl|Cbl
Developmental biology	0.16	P35235|P70424|Q01279|P26039|Q62077	Ptpn11|Erbb2|Egfr|Tln1|Plcγ1
**PyMT**			
**Type 2 diabetes mellitus**	**3.08E-02**	P35569|Q64143|Q63844|P26450|P28867	Irs1|Pik3r3|Mapk3|Pik3r1|Prkcd
**FcεRI signalling pathway**	**0.03**	P28867|Q63844|P25911|Q64143|Q9Z1B7|P26450	Prkcd|Mapk3|Lyn|Pik3r3|Mapk13|Pik3r1
**Acute myeloid leukaemia**	**0.03**	Q64143|Q63844|P05532|P26450|P42227	Pik3r3|Mapk3|Kit|Pik3r1|Stat3
**Fcγ receptor–mediated phagocytosis**	**0.04**	P28867|Q63844|P08103|Q64143|P25911|P26450	Prkcd|Mapk3|Hck|Pik3r3|Lyn|Pik3r1
**Neurotrophin signalling pathway**	**0.04**	P35569|P28867|Q63844|Q64143|Q9Z1B7|Q9QYY0|P26450	Irs1|Prkcd|Mapk3|Pik3r3|Mapk13|Gab1|Pik3r1
**Aldosterone-regulated sodium reabsorption**	**0.04**	P35569|Q64143|Q63844|P26450	Irs1|Pik3r3|Mapk3|Pik3r1
ErbB signalling pathway	0.09	Q9QYY0|Q63844|P26450|Q61526|Q64143	Gab1|Mapk3|Pik3r1|Erbb3|Pik3r3
Cell–cell communication	0.10	Q80W68|P30999|P26450|Q9Z1B7	Kirrel|Ctnnd1|Pik3r1|Mapk13
Hepatitis C	0.10	Q63844|Q9Z0G9|Q64143|Q9Z1B7|P42227|P26450	Mapk3|Cldn3|Pik3r3|Mapk13|Stat3|Pik3r1
Chemokine signalling pathway	0.12	P28867|Q63844|P08103|Q64143|P42227|P25911|P26450	Prkcd|Mapk3|Hck|Pik3r3|Stat3|Lyn|Pik3r1

^a^PyMT: Polyoma virus middle T antigen. Significantly enriched terms are shown in bold.

**Figure 4:**
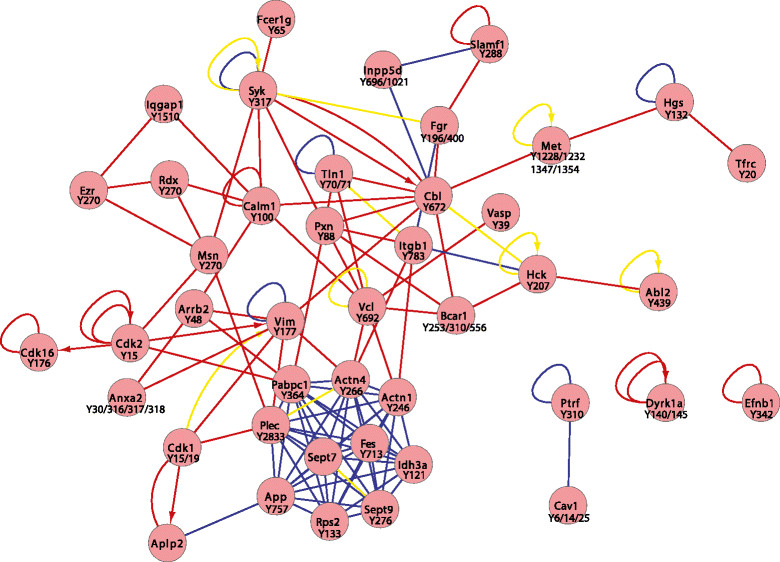
**Protein Interaction Network Analysis of proteins exhibiting significantly enhanced tyrosine phosphorylation in the p53 tumour model.** The direct protein–protein interactions are indicated by lines, with blue and red lines representing data from mouse and human orthologue databases, respectively. Interactions that have been described previously in both human and mouse are indicated by yellow lines. Kinase substrate relationships are indicated by arrows.

**Figure 5:**
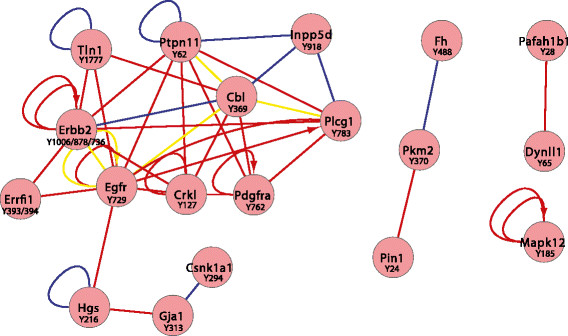
**Protein Interaction Network Analysis of proteins exhibiting significantly enhanced tyrosine phosphorylation in the Her2 tumour model.** The protein–protein interactions are indicated as described for Figure 4.

**Figure 6:**
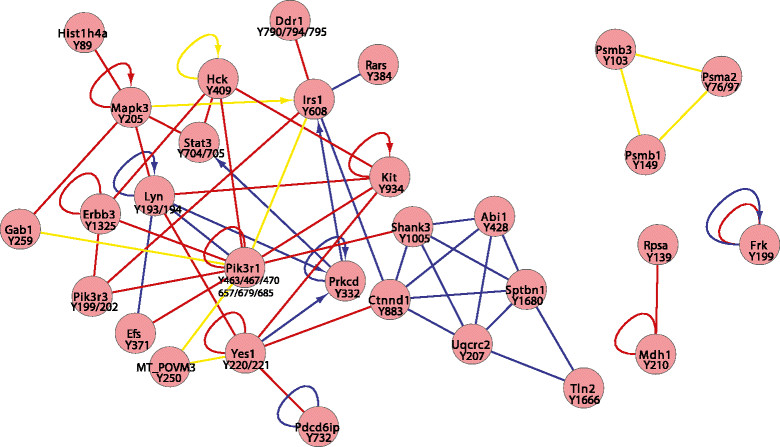
**Protein Interaction Network Analysis for proteins exhibiting significantly enhanced tyrosine phosphorylation in the polyoma virus middle T antigen tumour model.** The protein–protein interactions are indicated as described for Figure 4.

Significantly upregulated pY sites (determined by ANOVA) characteristic of each tumour type were analysed using PINA [37],[38] (Figures 4, 5 and 6). Where available, consensus lists were searched against mouse data sets containing previously reported interaction data; however, in cases where interactions were not reported in mouse data sets, human orthologues were used to model potential interactions. In addition, kinase substrate relationships were extracted from PhosphoSitePlus [39]. A key characteristic of p53 tumours was the presence of a major protein–protein interaction hub composed of several proteins involved in cytoskeletal organisation (Actn1, Actn4, Sept7, Sept 9 and Plec), as well as a further hub centred on Itgb1 (Figure 4). The latter involved numerous proteins localised to focal adhesions, including Bcar1, Tln1, Pxn and Vcl. These findings are in agreement with the corresponding pathway analysis (Table 1). Furthermore, there are notable similarities between this network and that present in human basal and claudin-low breast cancer cell lines [16], including increased tyrosine phosphorylation of Met, Cav1, Bcar1 and Tln1. This is consistent with previous reports that subsets of p53 tumours exhibit gene expression signatures characteristic of these breast cancer subgroups [21],[22]. In contrast, HER2 tumours exhibited a protein–protein interaction network that reflected the relationship between HER2 and its associating substrates (for example, Cbl, Ptpn11, Plcγ1), but it is noteworthy that Egfr and Pdgfrα, as well as the feedback inhibitor Errfi1, are also components of this network (Figure 5). Consistent with the pathway analysis, PyMT tumours exhibit a major interaction hub that centres on the PI3K regulatory subunit Pik3r1 (Figure 6). Interestingly, in addition to PyMT itself, this hub involves two RTKs (Kit and Erbb3) and two docking proteins (Irs1 and Gab1) that are well-characterised binding partners for Pik3r1. Although Src is required for efficient induction of mammary tumours by PyMT [50], increased phosphorylation of Y416 on the Src activation loop (a peptide sequence conserved amongst SFKs) or Src-selective peptides was not detected in PyMT tumours. However, three additional SFKs—Yes, Lyn and Hck—exhibited increased phosphorylation on unique peptide sequences and resided within the network (Figure 6). The former is known to interact with PyMT [19] but is not essential for PyMT-induced mammary tumourigenesis [50].

### Subclassification of p53 tumours

**Figure 7:**
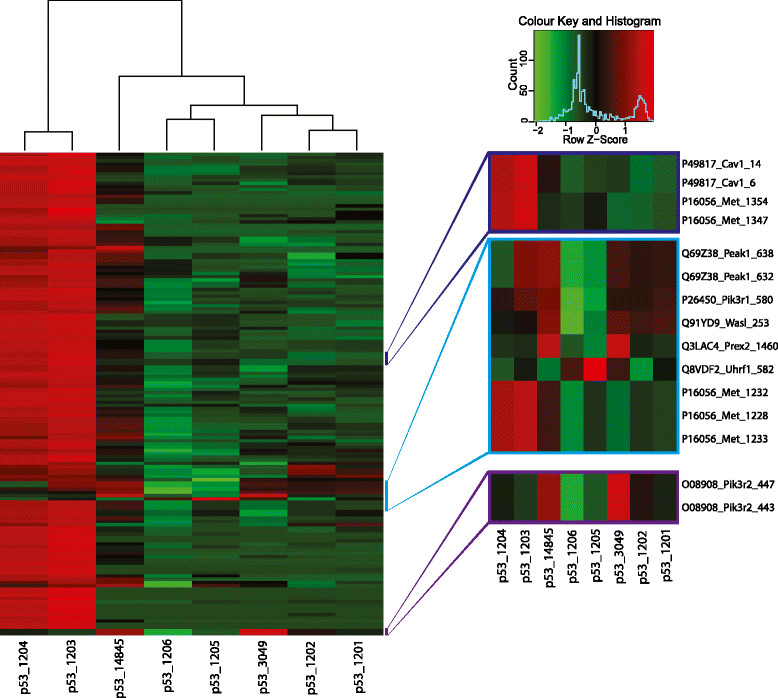
**Unsupervised hierarchical clustering using the 150 most variable phosphotyrosine sites identified in the p53 tumours.**

**Figure 8:**
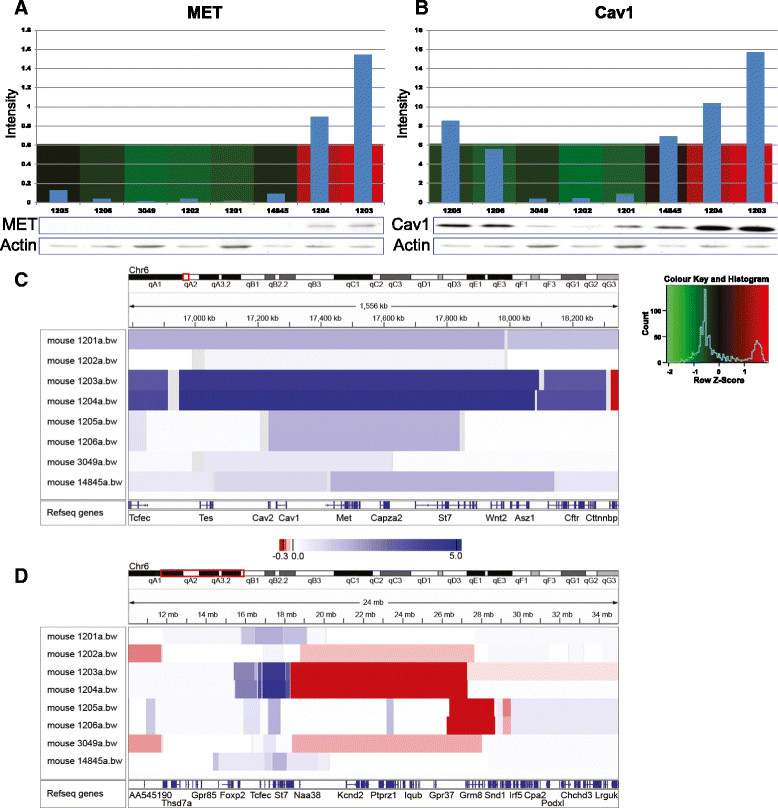
**Protein expression, tyrosine phosphorylation and gene copy number for Met and Cav1. (A)** and **(B)** Immunoblot quantitation of total protein expression compared with relative tyrosine phosphorylation of the corresponding protein obtained by mass spectrometry. Data are presented as in Figure 2. Colour key and histogram apply to **(A)** and **(B)**. The heat maps indicate the intensity of phosphorylation on the following specific sites: MET (pY1354) and Cav1 (pY14). **(C)** Array comparative genomic hybridisation profile of mouse chromosome 6 in the different p53 tumours, showing the 1,556-kb stretch where the genes for Cav1 and Met are located. **(D)** Lower-resolution view of the amplified region. The intensity of blue or red indicates gene copy number. Heat map in **(C)** also applies to **(D)**.

In a previous study on the Tp53-null mouse mammary tumour model, researchers demonstrated that the generated tumours exhibit considerable heterogeneity and can be classified by transcript profiling into a variety of molecular subtypes, including one luminal, one claudin-low and two basal-like subtypes [22]. To interrogate this potential heterogeneity at the level of tyrosine phosphorylation patterns, we undertook unsupervised hierarchical clustering of the p53 tumours based on all phosphorylation sites detected in these tumours (Additional file 8: Figure S6). Tumours 1203 and 1204 clustered together and were characterised by markedly elevated (greater than tenfold) tyrosine phosphorylation of approximately 100 proteins compared to the remainder of p53 tumours (Additional file 9: Table S3). Tumour 14845 also exhibited relatively high tyrosine phosphorylation of many proteins, but not to the same extent as tumours 1203 and 1204, and tumours 3049, 1201 and 1202 displayed elevated phosphorylation of more distinct protein subsets. Tumours 1205 and 1206 were characterised by relatively low levels of tyrosine phosphorylation. Hypothesizing that the extremely high levels of tyrosine phosphorylation in tumours 1203 and 1204 might reflect upregulation of a particular tyrosine kinase in these two tumours, we noted that several of the upregulated pY sites were from Met, specifically Y1228, Y1232, Y1233, Y1347 and Y1354 (Figure 7). In addition, Y6 and Y14 on Cav1 also exhibited increased phosphorylation, which was of interest, given the close proximity of the *Met* and *Cav1* genes on mouse chromosome 6 and coamplification of these two genes in particular tumour types [51]-[53]. Immunoblotting for Met and Cav1 revealed markedly increased total protein expression in the p53 samples from tumours 1203 and 1204 (Figure 8A and B). In addition, aCGH was performed on the p53 tumours. This revealed that, though p53 tumours 1201, 1205, 1206 and 14845 exhibited small (two- to threefold) increases in copy number for regions of chromosome 6 harbouring Met and Cav1, tumours 1203 and 1204 displayed 20- and 25-fold amplifications, respectively, of a chromosomal segment spanning these two genes (Figure 8C and D and Additional file 10: Table S4).

Having demonstrated that tumours 1203 and 1204 are characterised by Met gene amplification, we compared the tyrosine phosphorylation profile of these two tumours versus the other p53-null tumours in more detail. Consistent with enhanced Met signalling, tyrosine phosphorylation of Hgs, which regulates Met endocytic trafficking [54], was markedly elevated (Additional file 9: Table S3). These tumours were also characterised by increased tyrosine phosphorylation of Ptrf/cavin, which is localised with Cav1 to caveolae, and of the RTK Axl and the docking protein Dock1 (Additional file 9: Table S3). Interestingly, pathway analysis revealed that tumours 1203/1204 demonstrated significant enrichment for the term glycolysis/gluconeogenesis, reflecting increased tyrosine phosphorylation of several metabolic enzymes, including Pgam1, Pkm2 and Pgk1 (Additional file 11: Table S5).

**Figure 9:**
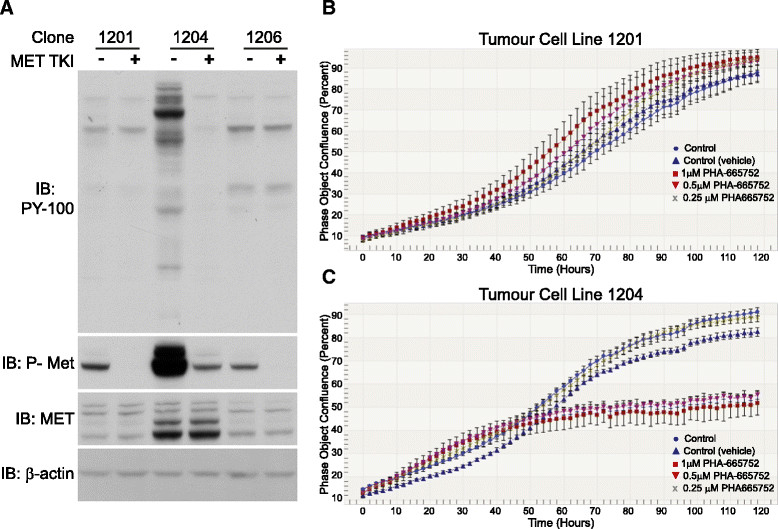
**Effects of MET inhibition on p53-null tumour-derived cell lines. (A)** Effect of the Met tyrosine kinase inhibitor (TKI) PHA-665752 on cellular tyrosine phosphorylation in different p53-null tumour-derived cell lines. Cell lysates were subjected to Western blot analysis as indicated. **(B)** and **(C)** Effect of Met inhibition on proliferation of cells with low (tumour cell line 1201, **B)** and high (tumour cell line 1204, **C)** levels of Met gene amplification. IB, Immunoblot.

To gain insight into the functional role of Met in the p53 tumours, we established cell lines from tumours 1201 and 1206 (low-level Met gene amplification) and tumour 1204 (high-level Met amplification). Pretreatment of these cell lines with the Met TKI PHA-665752 decreased Met phosphorylation on Y1234/Y1235 and markedly reduced the elevated cellular tyrosine phosphorylation levels in tumour 1204 cells (Figure 9A). Importantly, though administration of the Met TKI at a concentration (1 μM) that ablated Met tyrosine phosphorylation had no effect on proliferation in tumour 1201 cells (Figure 9B), this treatment markedly attenuated proliferation of tumour 1204 cells (Figure 9C). As an alternative and complementary approach, we knocked down Met expression using selective siRNAs (Additional file 12: Figure S7). Met knockdown had only a modest effect in the 1201 and 1206 tumour cell lines, but markedly attenuated proliferation in the 1204 tumour cell line with high-level Met amplification (*P* < 0.005 for comparisons of Met knockdowns in the 1201 or 1206 tumour cell line versus the 1204 tumour cell line). These data indicate that the extremely high levels of tyrosine phosphorylation detected in tumour 1204 (Figure 7) are dependent on Met activity, and that cells from these tumours are addicted to Met activation for their proliferation *in vitro*.

Interrogation of the tyrosine phosphorylation patterns characteristic of the other p53-null subgroups revealed that phosphorylation of the atypical kinase Peak1/SgK269 [55] was elevated in tumour 14845 and to a lesser extent the subgroup containing 3049, 1202 and 1201, whereas phosphorylation of Prex2 characterised tumours 14845 and 3049, where it was accompanied by enhanced phosphorylation of Pik3r2 (Figure 7). Taken in association with our demonstration of Met amplification in tumours 1203 and 1204, these findings highlight the power of tyrosine phosphorylation profiling to identify candidate oncogenic drivers [56].

## Discussion

Specific genetically modified mouse models of breast cancer have made major contributions to our understanding of the role of particular oncogenes in the development of this malignancy [18]. In addition, characterisation of tumours arising in these models by approaches such as transcript profiling and aCGH have identified molecular aberrations conserved between mouse and human mammary tumours that are likely to represent key 'driver' events [21],[22],[57]. However, to date, no comprehensive characterisation of the tyrosine kinase signalling networks present in such models has yet been undertaken. In this study, we used MS-based global tyrosine phosphorylation profiling to characterise three commonly used mouse models of breast cancer, which revealed distinct differences between these models and features conserved with subtypes of the human disease.

For an erbB2 model, we used a truncated form of Neu [26],[27] that models a form of erbB2 lacking the extracellular domain present in approximately 30% of HER2-positive breast cancer patients, for whom it is associated with a worse prognosis [58]. This form exhibits potent oncogenic activity [59]. These tumours were characterised by increased phosphorylation of several signalling molecules known to associate with erbB2, including Ptpn11, Plcγ1, Cbl and CrkL, as well as its heterodimerisation partner Egfr. Given the known heterodimerisation of full-length erbB2 and erbB3, our finding that phosphorylation of erbB3 Y1325 was not elevated in the majority of HER2 tumours (expressing truncated neu), despite increased erbB3 expression (Figure 2), is surprising and warrants further investigation in cell culture models and clinical specimens. Additional novel findings include enhanced tyrosine phosphorylation of Errfi1 and Pdgfrα. The former represents a negative feedback regulator of erbB signalling that inhibits EGFR and erbB2 kinase activity by binding directly to the receptor kinase domain and blocking the formation of the activating dimer interface [60]. However, although increased expression of Errfi1 is associated with high erbB receptor signalling [61], expression of Errfi1 was similar between HER2 and p53 tumours, indicating that it is the relative tyrosine phosphorylation of Errfi1 on Y393/Y394 that is altered. Importantly, phosphorylation of Errfi1 on Y394 reduces its ability to inhibit Egfr [62], indicating that this modification may act to attenuate negative regulation of HER2 signalling by Errfi1 in these tumours. Consequently, it will be of great interest to determine how expression of Errfi1, and also its tyrosine phosphorylation, relate to HER2 expression and outcome in breast cancer patients.

The finding of increased Pdgfrα phosphorylation is of interest because autocrine Pdgf action is associated with TGF-β-induced epithelial–mesenchymal transition (EMT) in MMTV-Neu mammary tumours and because increased Pdgfrα and Pdgfrβ expression occur in late-stage, invasive human breast cancers [63]. Researchers in a previous study demonstrated that a truncated form of HER2, similar to that used in this study, specifically induced the expression of a set of genes involved in cancer cell spread and associated with poor patient prognosis [59]. Thus, the induction of Pdgfrα signalling in our model may reflect the oncogenic potency of the truncated form of Neu utilised and the occurrence of EMT.

A previous study on the PyMT model revealed a dependence on PI3K activation for efficient tumourigenesis [20]. Although that finding was revealed by mutation of the p85 binding sites on PyMT [20], our pathway and protein–protein interaction analyses on the tyrosine-phosphorylated proteins selectively enriched in these tumours also support a key role for PI3 kinase signalling in this tumour type. These results highlight the presence of other p85 binding proteins in these tumours in addition to PyMT itself, including erbB3, Gab1 and Irs1. All of the latter three proteins have been characterised as positive regulators of PI3K signalling [64]. In addition, enhanced tyrosine phosphorylation of p85a on several sites was detected in these tumours. Tyrosine phosphorylation of p85a on Y688 has been reported to relieve its inhibitory activity on PI3K [65]; however, we did not detect phosphorylation on this site in our present study, and the functional role of the p85a phosphorylation events enhanced in PyMT tumours requires further characterisation.

An important question is the identity of the tyrosine kinases driving PyMT tyrosine phosphorylation. It is well established that PyMT associates with, and is phosphorylated by, the SFKs Src and Yes, and also that expression of Src is essential for efficient PyMT-induced tumourigenesis [19],[50]. However, in a recent article, researchers reported that PyMT associates with the RTK erbB2 and the catalytically impaired erbB3 and that both erbB2 activity and erbB3 expression are essential for PyMT transforming activity [66]. These data, together with those published by the Muller group [50], indicate that erbB2 may act upstream of Src in this model. However, we could detect only erbB3-derived, and not erbB2-derived, tyrosine-phosphorylated peptides in the majority of PyMT tumours. A potential explanation is that one or more RTKs may substitute for erbB2 in this model. Because Egfr phosphorylation was detected and phosphorylation of Kit was enhanced in this tumour type, these RTKs represent potential candidates.

The p53 tumour model exhibits heterogeneity at the histological and gene expression levels, and, on the basis of the latter parameter, can be classified into different molecular subgroups, including basal-like, claudin-low and luminal subtypes [22]. Although we profiled only eight p53 tumours, our data are consistent with the ability of this model to generate tumours with a basal or claudin-low phenotype. First, there was notable overlap between the identified signalling networks in these tumours and basal and claudin-low breast cancer cells [16]. Second, tyrosine-phosphorylated proteins characteristic of p53 tumours exhibited an enrichment for pathways associated with cytoskeletal organisation, in concordance with the observed mutational spectrum reported for triple-negative breast cancers [67]. Third, two of the p53 tumours exhibited markedly enhanced tyrosine phosphorylation of many cellular proteins, including Met, and this was associated with high-level amplification of a segment of chromosome 6 that contains the *Met* and *Cav1* genes. This is consistent with high expression and amplification of the *MET* gene in human basal breast cancers [13],[15],[68] and amplification of the Met/Cav1 locus in basal-like mammary tumours in mice with mammary-specific deletion of Lfng [52]. In addition, researchers in a recent study demonstrated that transgenic Met overexpression in the mouse mammary gland cooperates with conditional p53 loss to drive the formation of claudin-low mammary tumours, and they showed that claudin-low tumours developing in mice without the MMTV-Met transgene and only conditional mammary gland-specific deletion of p53 exhibited amplification of the endogenous *Met* gene [69]. These data are in concordance with our data demonstrating Met amplification in a subset of p53-null mammary tumours and highlight the potential utility of transplantable tumours 1203 and 1204 and the MMTV-Met^mt^;Trp53fl/+;Cre model generated by the Park group [69] as preclinical models in which to test Met-directed therapies for triple-negative breast cancer. Indeed, our data indicate that p53-null cells with high-level Met amplification are dependent on Met activation for their continued proliferation.

Our finding that tumours 1203 and 1204 demonstrated increased tyrosine phosphorylation of several enzymes involved in glycolysis and gluconeogenesis indicates that this tumour subset is likely to exhibit altered metabolic properties. Indeed, tyrosine phosphorylation of both Pkm2 Y105 and Pgam1 Y26 regulates their activity and, by modulating aerobic glycolysis and anabolic biosynthesis, promotes tumourigenesis [70],[71]. In light of these findings, it will be important to determine whether MET-amplified human breast cancers exhibit corresponding metabolic changes and whether the altered metabolism confers an 'Achilles heel' amenable to therapeutic intervention.

The identification of two p53 tumours exhibiting *Met* gene amplification highlights how mouse models of mammary tumourigenesis can be used to identify breast cancer oncogenic 'drivers'. Of note, our phosphoproteomic profiling of p53 tumours identified markedly enhanced tyrosine phosphorylation of Peak1/SgK269 and Prex2 in other p53-null tumour subsets. The former protein is an atypical kinase recently demonstrated to exhibit characteristics of a breast cancer oncogene, and site-selective Peak1/SgK269 tyrosine phosphorylation promotes biological activity [55]. Prex2 is a negative regulator of PTEN (phosphatase and tensin homologue) and is mutated in melanoma [72], although the role of Prex2 tyrosine phosphorylation is yet to be determined. Consequently, these two proteins represent strong candidates for further functional characterisation and evaluation as therapeutic targets and biomarkers.

## Conclusions

Our finding that three commonly utilised mouse models of breast cancer are characterised by distinct tyrosine phosphorylation–based signalling networks provides important insights into mechanisms of mammary tumour development and progression. The identification of Met as an oncogenic driver in a subset of p53-null tumours highlights how comparative phosphoproteomics can identify conserved oncogenic signalling pathways and identifies a new preclinical model for a subset of triple-negative breast cancers.

## Additional files

## Electronic supplementary material

Additional file 1: Figure S1.: **(A)** The lineage of the different tumour samples characterised. **(B)** Clustering relationships in comparison to tumour of origin for the xenografts. The coloured lines indicate which tumour transplants share a common origin. The coloured ovals indicate instances where only one transplant tumour from a particular clonal line was characterised. (PDF 123 KB)

Additional file 2: Table S1.: 763 pY sites identified from the pY profiling of p53, Her2 and PyMT mammary mouse tumours. (XLSX 166 KB)

Additional file 3: Figure S2.: **(A)** Gene Ontology annotation of the phosphoproteins identified from the three mouse tumour models. **(B)** Proportion of different kinase types identified (based on number and subclassification). **(C)** Contribution of different non-receptor tyrosine kinases, based on pY peptide spectral intensity. **(D)** Contribution of different receptor tyrosine kinases based on pY peptide spectral intensity. (PDF 1 MB)

Additional file 4: Figure S3.: Unsupervised hierarchical clustering using all 763 identified pY sites. (Imputation: *k*-NN + column mean). (PDF 245 KB)

Additional file 5: Table S2.: 381 highly reproducible pY sites. (XLSX 122 KB)

Additional file 6: Figure S4.: Unsupervised hierarchical clustering using the 381 pY sites detected in at least 75% of one tumour type. (Imputation: *k*-NN+ row min). (PDF 175 KB)

Additional file 7: Figure S5.: **(A)** and **(B)** Immunoblot quantitation of phosphorylation on specific phosphosites compared with relative tyrosine phosphorylation on the same sites determined by MS. Data are presented as in Figure 2, and colour key and histogram apply to (A) and (B). The heat maps indicate the intensity of phosphorylation on the following specific sites: ErbB3 (pY1325) and p85 (pY467). The lane corresponding to tumour 1186 has been removed due to sample degradation. Because phosphorylation of Y1325 was not detected in HER2 tumour 3051 by MS, the positive signal obtained by Western blotting may reflect cross-reactivity of the commercial antibody with a different phosphorylation site on erbB3 or another erbB receptor. (PDF 611 KB)

Additional file 8: Figure S6.: Unsupervised hierarchical clustering using the 707 pY sites identified in p53 tumours. (Imputation: *k*-NN + column mean). (PDF 149 KB)

Additional file 9: Table S3.: Proteins showing >10-fold increased phosphorylation in 1203/4 versus the other p53 tumours. (XLSX 103 KB)

Additional file 10: Table S4.: Amplified regions on chromosome 6 for the eight p53 tumours. (XLSX 13 KB)

Additional file 11: Table S5.: Pathway analysis of proteins showing >10-fold increased phosphorylation in 1203/4 versus other p53 tumours. (XLSX 23 KB)

Additional file 12: Figure S7.: **(A)** MET knockdown using siRNA in cell lines derived from p53-null mouse tumours. Western blots were incubated with the indicated antibodies. # in 1201 indicates nonspecific band. **(B)** Effect of MET knockdown on cell proliferation by MTS assay measuring relative absorbance. At least three independent sets of replicate experiments were performed with the same trend observed. Data from one representative experiment are shown. *P < 0.05 and **P < 0.005 by *t*-test (siRNA treated vs control). 'Control' refers to ON-TARGETplus nontargeting siRNA, and 'Pool' refers to SMARTpool consisting of four individual siRNAs targeting mouse MET. (PDF 947 KB)

Below are the links to the authors’ original submitted files for images.

Authors’ original file for figure 1

Authors’ original file for figure 2

Authors’ original file for figure 3

Authors’ original file for figure 4

Authors’ original file for figure 5

Authors’ original file for figure 6

Authors’ original file for figure 7

Authors’ original file for figure 8

Authors’ original file for figure 9

## Abbreviations

aCGH: Array comparative genomic hybridisation
ANOVA: Analysis of variance
EMT: Epithelial–mesenchymal transition
FDR: False discovery rate
GEM: Genetically engineered mouse
Gprc5c: G protein–coupled receptor family C, group 5, member C
IP: Immunoprecipitation
*k*-NN *k*: -nearest neighbour
MMTV: Mouse mammary tumour virus
MS/MS: Tandem mass spectrometry
nano-LC-MS/MS: Nano-liquid chromatography tandem mass spectrometry
PI3K: Phosphatidylinositol 3-kinase
PINA: Protein interaction network analysis
pY: Phosphotyrosine
PyMT: Polyoma virus middle T antigen
RTK: Receptor tyrosine kinase
SFK: Src family kinase
