# Supplementary figures and images for: Profiling the tyrosine phosphoproteome of different mouse mammary tumour models reveals distinct, model-specific signalling networks and conserved oncogenic pathways
Source: Breast Cancer Res. 2014 Sep 9;16:437. doi: 10.1186/s13058-014-0437-3 (PMC4303118; doi:10.1186/s13058-014-0437-3)

**A**

| Tumor type/ original clone       | Xenograft - Tumor number |
|----------------------------------|--------------------------|
| <u><i>tp53</i><sup>-/-</sup></u> |                          |
| 117                              | 1204,1203                |
| 116                              | 1202,1201,3049           |
| 113                              | 1206,1205                |
| 5101 (BI6)                       | 14845                    |
| <u>Her2/Neu</u>                  |                          |
| 436                              | 1200,3050,3057           |
| 438                              | 3051,1197                |
| <u>PyMT</u>                      |                          |
| 10 No.2                          | 3075                     |
| 20 No.1                          | 3076, 3077               |
| 271                              | 3096,1186                |
| 20 No.2                          | 3095                     |
| 263                              | 1185                     |
| 264                              | 1187                     |

**B**

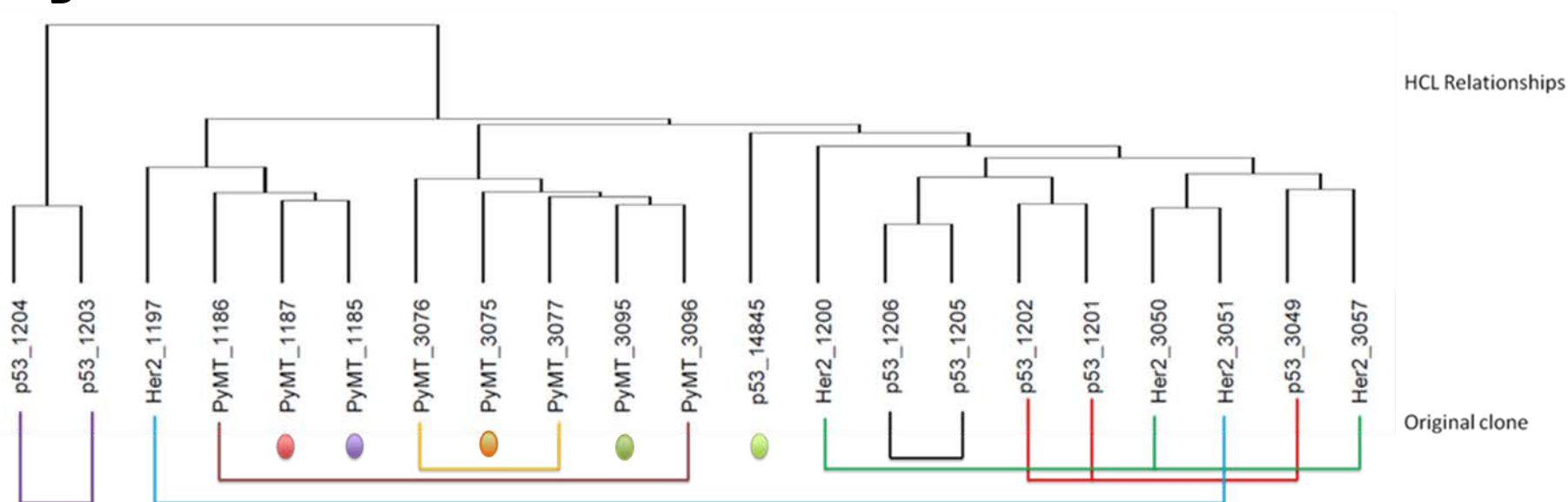

Supplement: Supplementary file 1 — Additional file 1: Figure S1.: (A) The lineage of the different tumour samples characterised. (B) Clustering relationships in comparison to tumour of origin for the xenografts. The coloured lines indicate which tumour transplants share a common origin. The coloured ovals indicate instances where only one transplant tumour from a particular clonal line was characterised. (PDF 123 KB) [file 13058_2014_437_MOESM1_ESM.pdf]

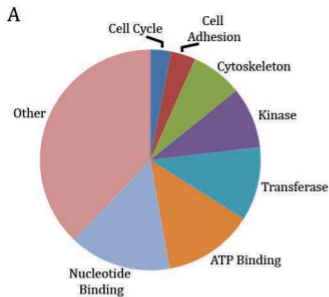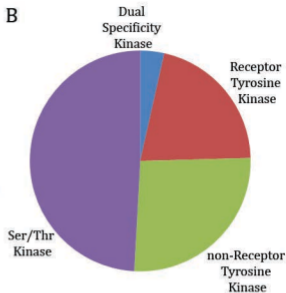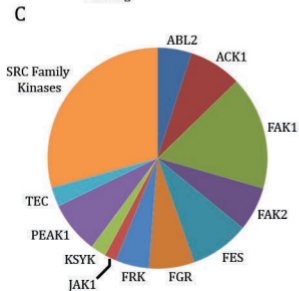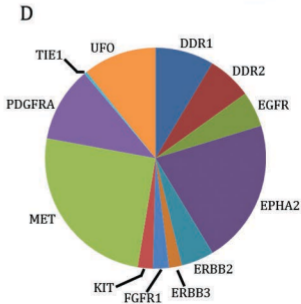

Supplement: Supplementary file 3 — Additional file 3: Figure S2.: (A) Gene Ontology annotation of the phosphoproteins identified from the three mouse tumour models. (B) Proportion of different kinase types identified (based on number and subclassification). (C) Contribution of different non-receptor tyrosine kinases, based on pY peptide spectral intensity. (D) Contribution of different receptor tyrosine kinases based on pY peptide spectral intensity. (PDF 1 MB) [file 13058_2014_437_MOESM3_ESM.pdf]

**Color Key  
and Histogram**

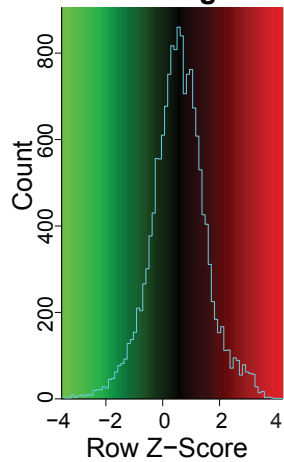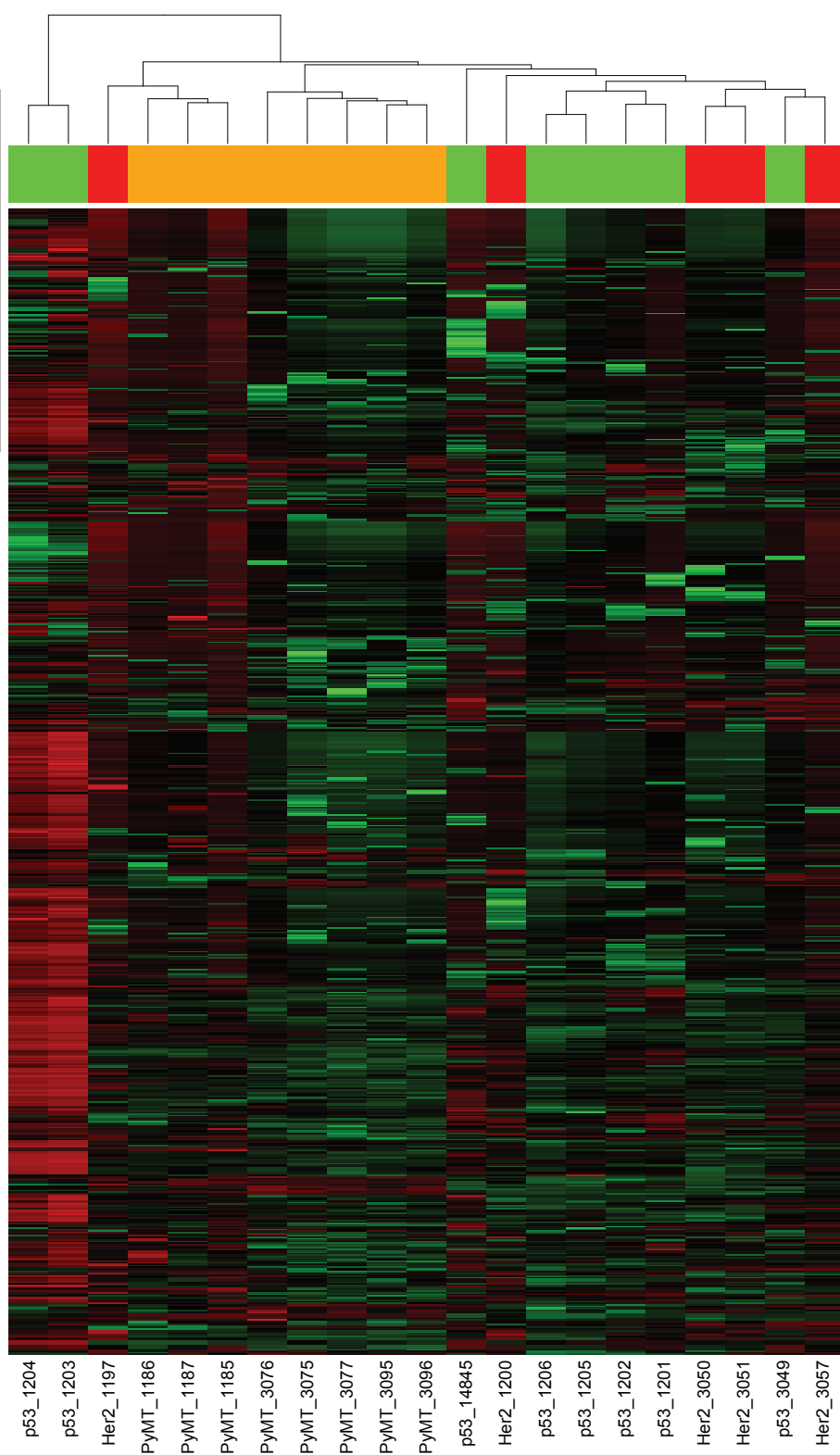

Supplement: Supplementary file 4 — Additional file 4: Figure S3.: Unsupervised hierarchical clustering using all 763 identified pY sites. (Imputation: k-NN + column mean). (PDF 245 KB) [file 13058_2014_437_MOESM4_ESM.pdf]

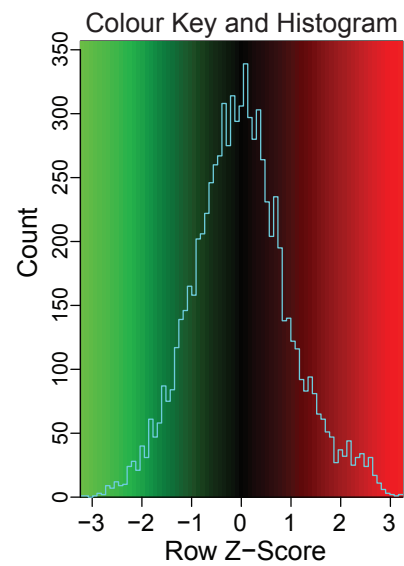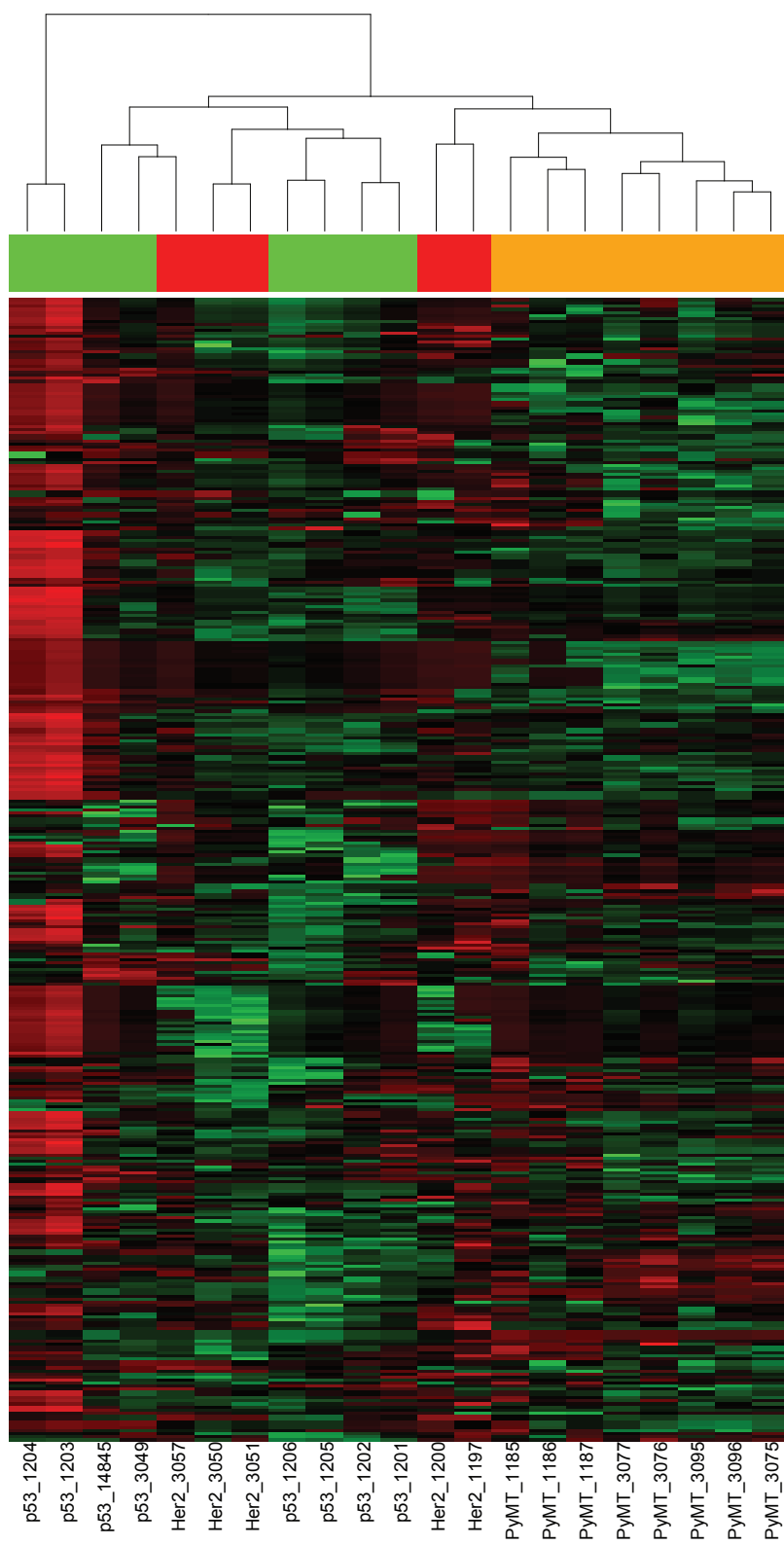

Supplement: Supplementary file 6 — Additional file 6: Figure S4.: Unsupervised hierarchical clustering using the 381 pY sites detected in at least 75% of one tumour type. (Imputation: k-NN+ row min). (PDF 175 KB) [file 13058_2014_437_MOESM6_ESM.pdf]

**A**

**ErbB3 (pY1325)**

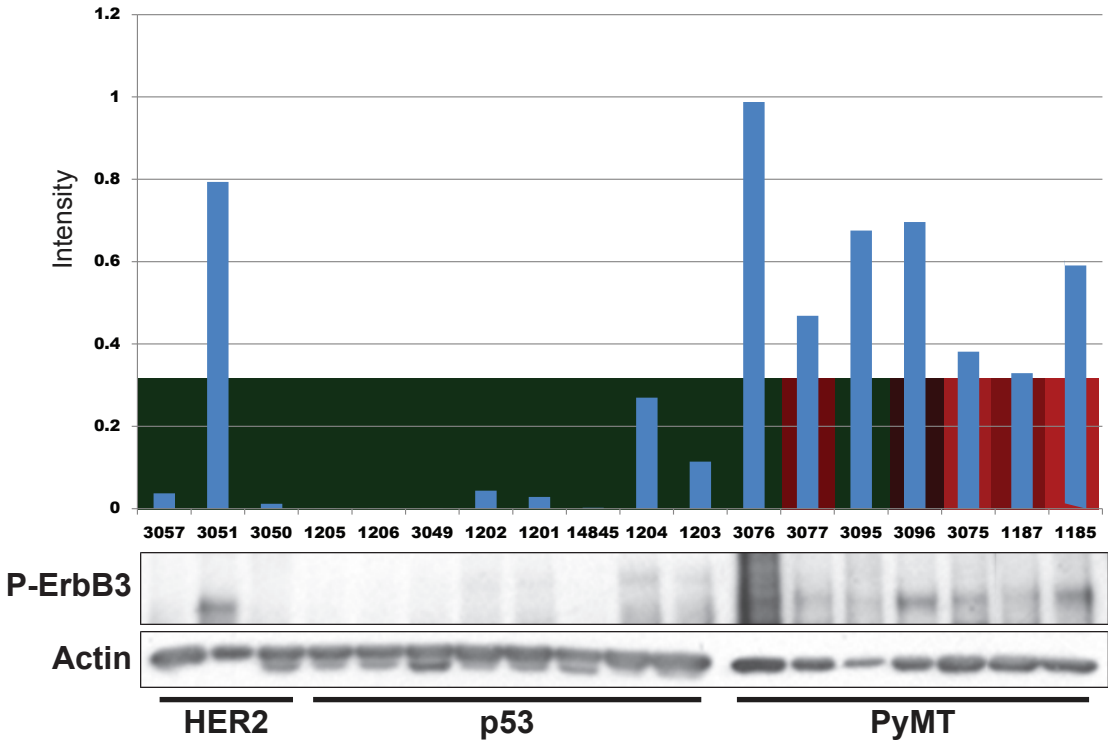

**B**

**p85 (pY467)**

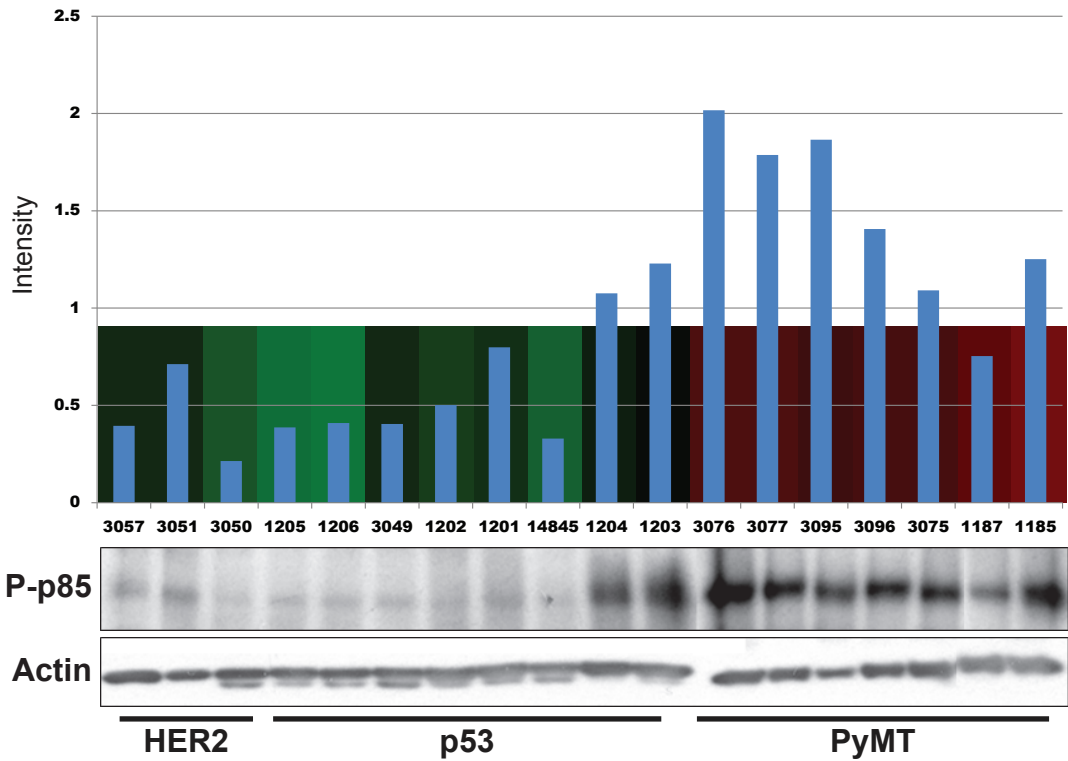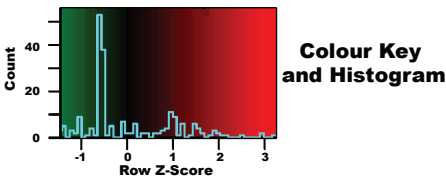

Supplement: Supplementary file 7 — Additional file 7: Figure S5.: (A) and (B) Immunoblot quantitation of phosphorylation on specific phosphosites compared with relative tyrosine phosphorylation on the same sites determined by MS. Data are presented as in Figure 2, and colour key and histogram apply to (A) and (B). The heat maps indicate the intensity of phosphorylation on the following specific sites: ErbB3 (pY1325) and p85 (pY467). The lane corresponding to tumour 1186 has been removed due to sample degradation. Because phosphorylation of Y1325 was not detected in HER2 tumour 3051 by MS, the positive signal obtained by Western blotting may reflect cross-reactivity of the commercial antibody with a different phosphorylation site on erbB3 or another erbB receptor. (PDF 611 KB) [file 13058_2014_437_MOESM7_ESM.pdf]

Colour Key and Histogram

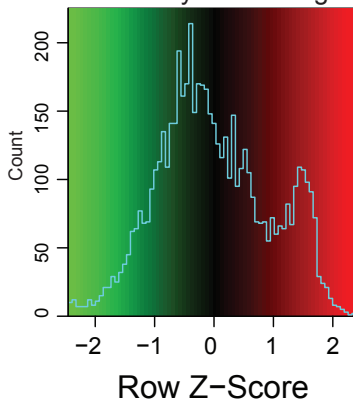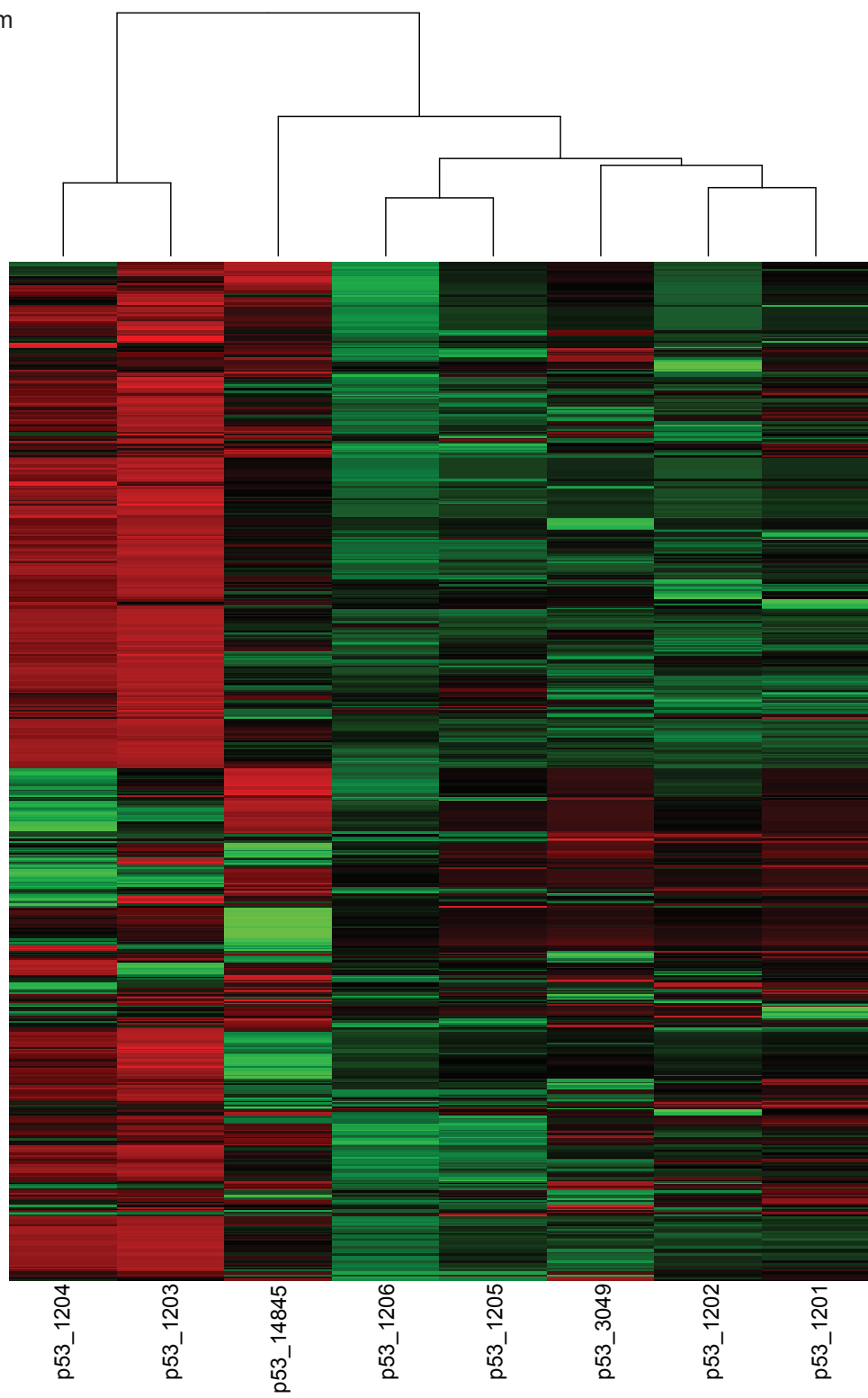

Supplement: Supplementary file 8 — Additional file 8: Figure S6.: Unsupervised hierarchical clustering using the 707 pY sites identified in p53 tumours. (Imputation: k-NN + column mean). (PDF 149 KB) [file 13058_2014_437_MOESM8_ESM.pdf]

**A**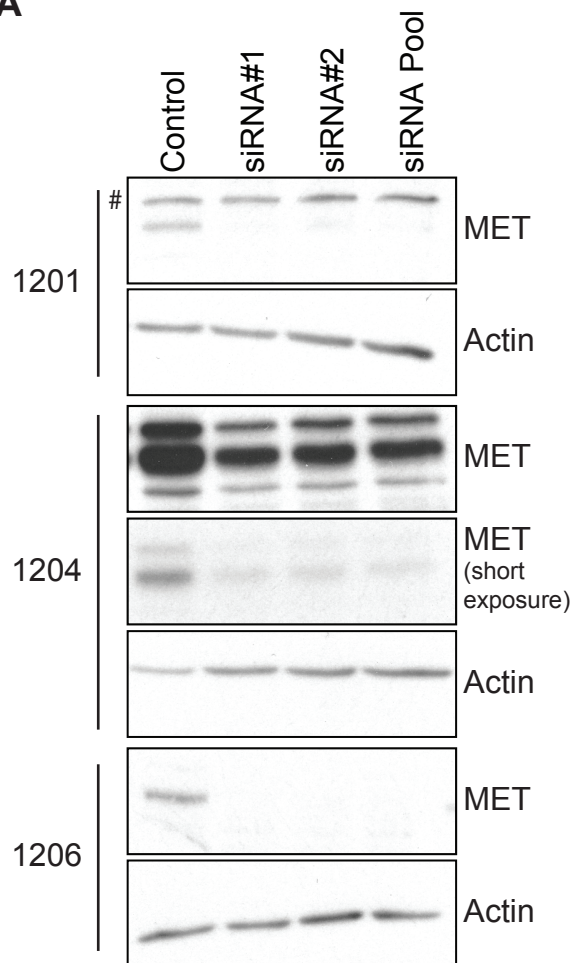**B**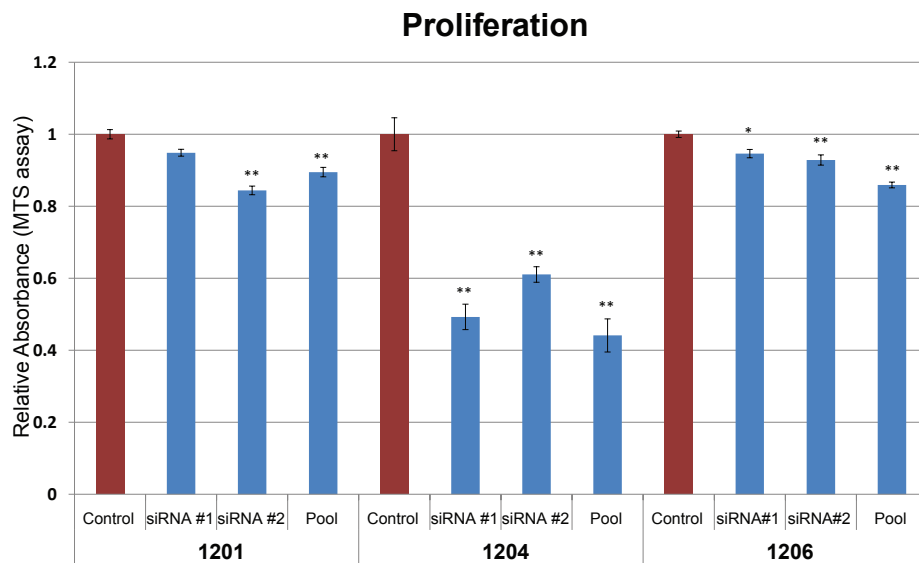

Supplement: Supplementary file 12 — Additional file 12: Figure S7.: (A) MET knockdown using siRNA in cell lines derived from p53-null mouse tumours. Western blots were incubated with the indicated antibodies. # in 1201 indicates nonspecific band. (B) Effect of MET knockdown on cell proliferation by MTS assay measuring relative absorbance. At least three independent sets of replicate experiments were performed with the same trend observed. Data from one representative experiment are shown. *P < 0.05 and **P < 0.005 by t-test (siRNA treated vs control). 'Control' refers to ON-TARGETplus nontargeting siRNA, and 'Pool' refers to SMARTpool consisting of four individual siRNAs targeting mouse MET. (PDF 947 KB) [file 13058_2014_437_MOESM12_ESM.pdf]

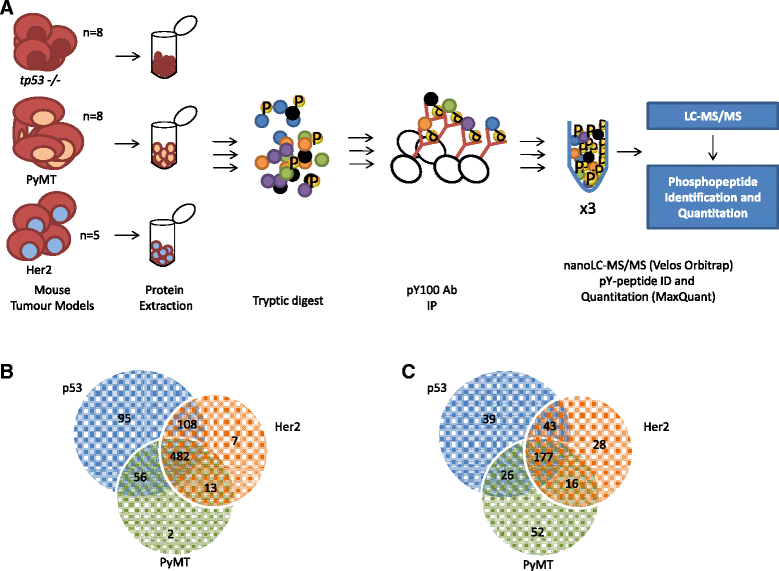

Supplement: Supplementary file 13 — Authors’ original file for figure 1 [file 13058_2014_437_MOESM13_ESM.gif]

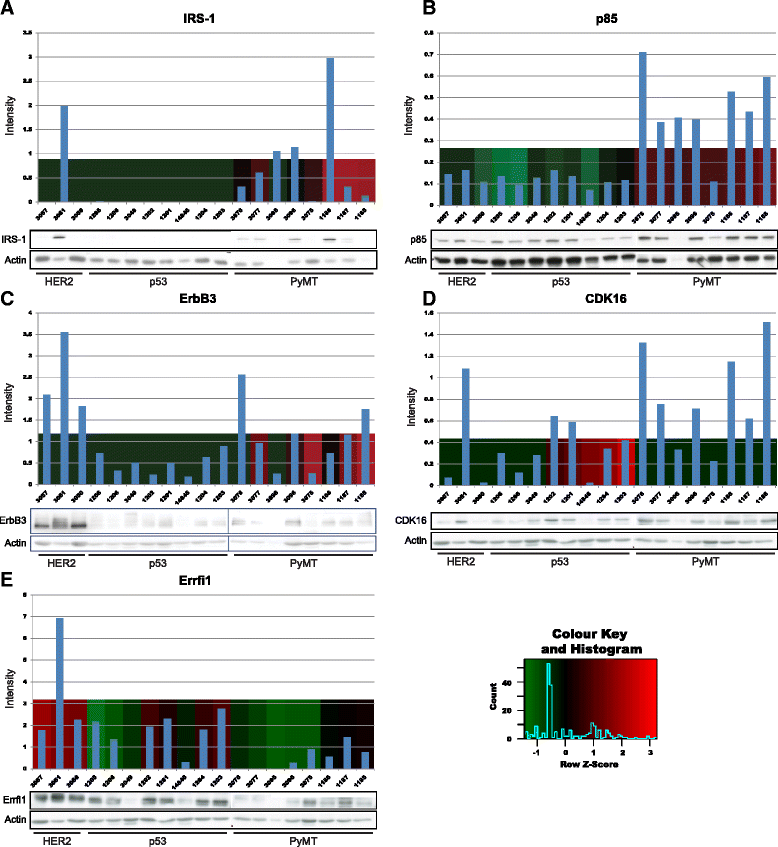

Supplement: Supplementary file 14 — Authors’ original file for figure 2 [file 13058_2014_437_MOESM14_ESM.gif]

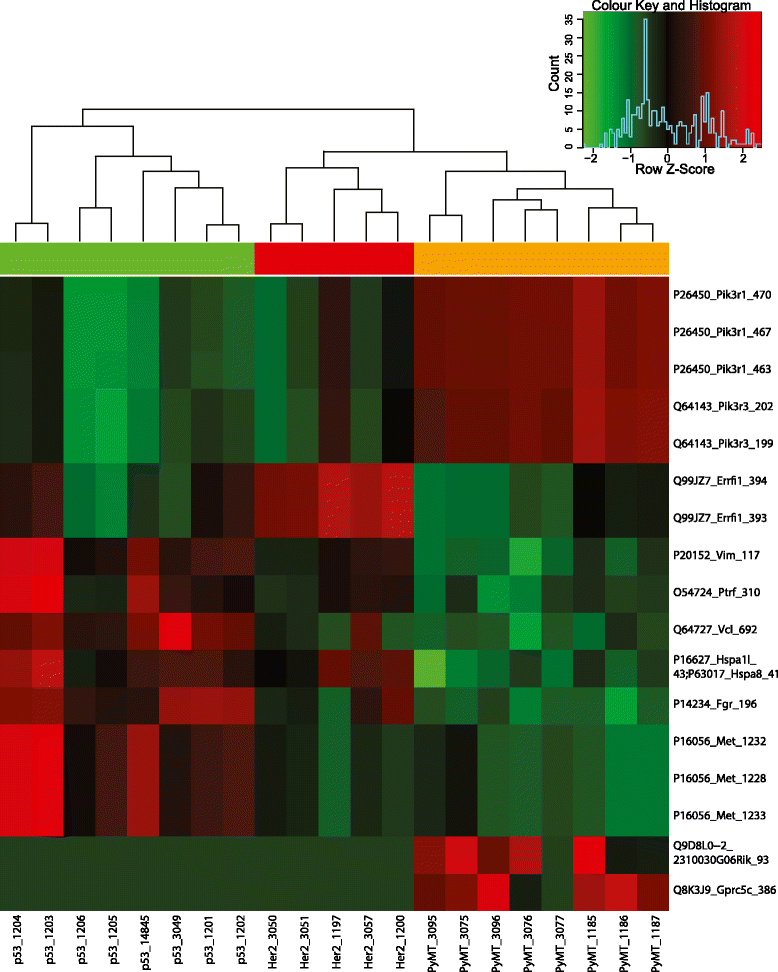

Supplement: Supplementary file 15 — Authors’ original file for figure 3 [file 13058_2014_437_MOESM15_ESM.gif]

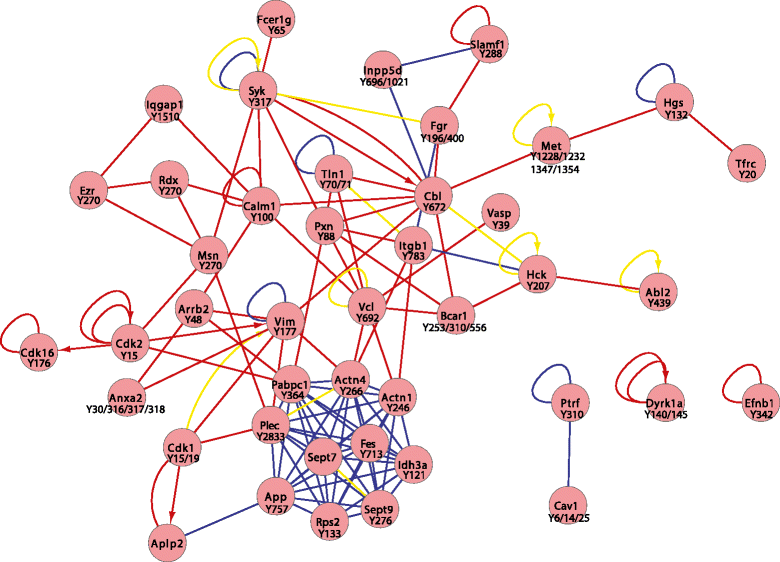

Supplement: Supplementary file 16 — Authors’ original file for figure 4 [file 13058_2014_437_MOESM16_ESM.gif]

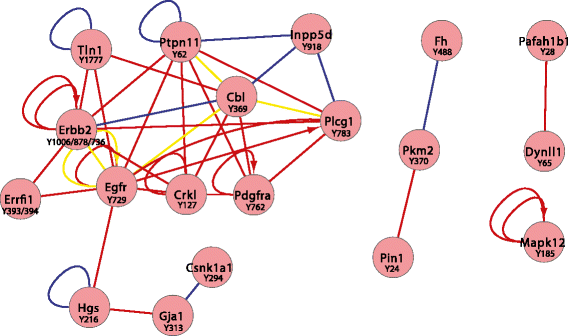

Supplement: Supplementary file 17 — Authors’ original file for figure 5 [file 13058_2014_437_MOESM17_ESM.gif]

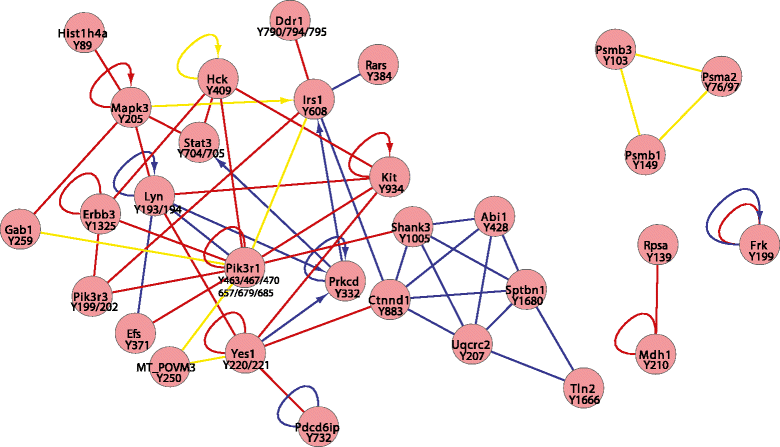

Supplement: Supplementary file 18 — Authors’ original file for figure 6 [file 13058_2014_437_MOESM18_ESM.gif]

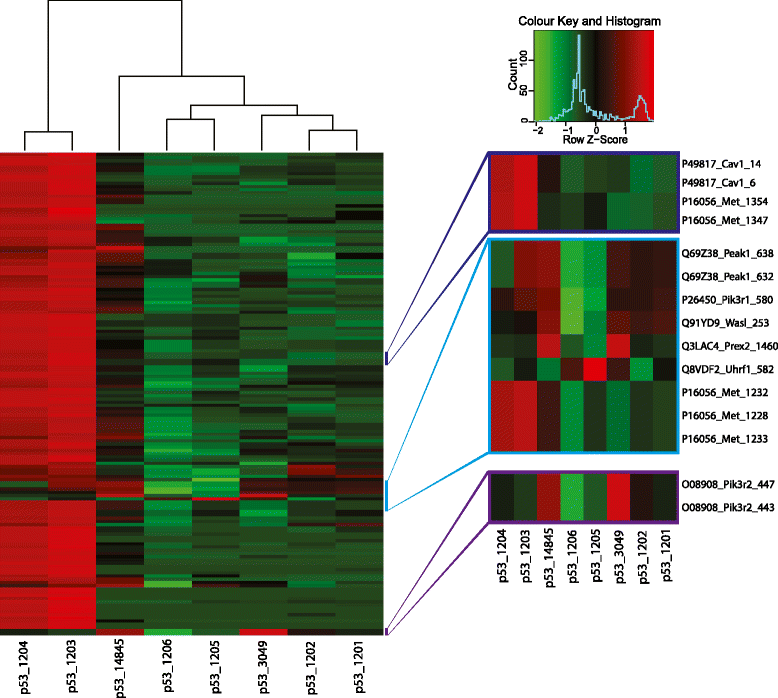

Supplement: Supplementary file 19 — Authors’ original file for figure 7 [file 13058_2014_437_MOESM19_ESM.gif]

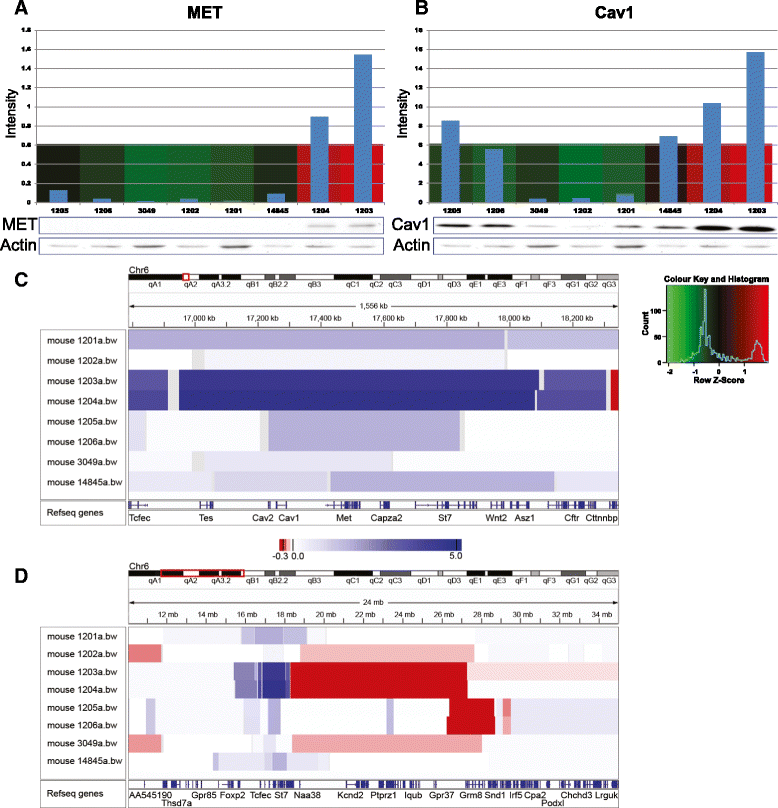

Supplement: Supplementary file 20 — Authors’ original file for figure 8 [file 13058_2014_437_MOESM20_ESM.gif]

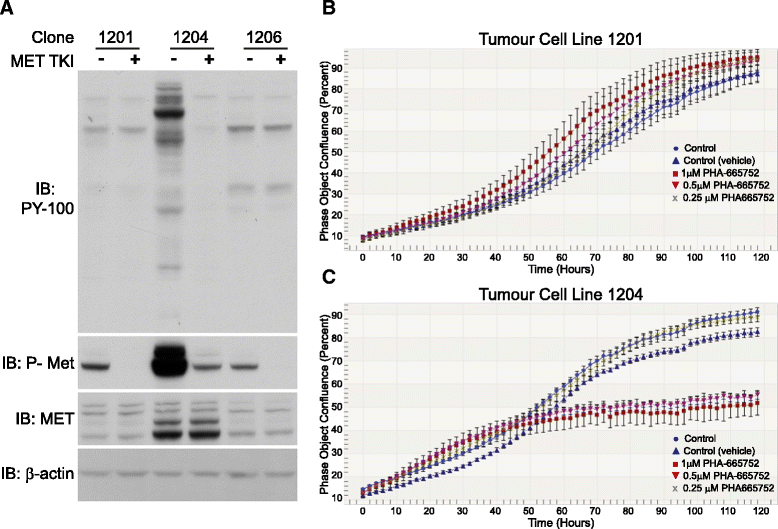

Supplement: Supplementary file 21 — Authors’ original file for figure 9 [file 13058_2014_437_MOESM21_ESM.gif]
